# ﻿Exploring *Monacha* species from the island of Corfu (NW Greece) by an integrative approach: new insights on *M. claustralis* (Rossmässler, 1834), *M. parumcincta* (Rossmässler, 1834) and allied species (Gastropoda, Eupulmonata, Hygromiidae)

**DOI:** 10.3897/zookeys.1250.159585

**Published:** 2025-08-28

**Authors:** Giuseppe Manganelli, Joanna R. Pieńkowska, Debora Barbato, Andrea Benocci, Katarzyna Sosnowska, Roy Anderson, Folco Giusti, Andrzej Lesicki

**Affiliations:** 1 Dipartimento di Scienze Fisiche, della Terra e dell'Ambiente, Università di Siena, Via Mattioli 4, 53100 Siena, Italy Università di Siena Siena Italy; 2 NBFC (National Biodiversity Future Center), Piazza Marina 61, 90133 Palermo, Italy NBFC (National Biodiversity Future Center) Palermo Italy; 3 Department of Cell Biology, Institute of Experimental Biology, Faculty of Biology, Adam Mickiewicz University in Poznan, Uniwersytetu Poznańskiego 6, 61-614 Poznań, Poland Adam Mickiewicz University in Poznan Poznań Poland; 4 Museo di Storia Naturale dell'Accademia dei Fisiocritici, Piazzetta S. Gigli 2, 53100 Siena, Italy Museo di Storia Naturale dell’Accademia dei Fisiocritici Siena Italy; 5 1 Belvoirview Park, Belfast, N. Ireland, BT8 7BL, UK Unaffiliated Belfast United Kingdom

**Keywords:** Allometry, genitalia, LDA Ratio Extractor, morphometry, nucleotide sequences, PCA Ratio Spectrum, phylogeography, shell

## Abstract

The Greek island of Corfu (Kérkyra) is considered the type locality of two *Monacha* species described in 1834 by Rossmässler, namely *Monacha claustralis* and *M. parumcincta*. In this work, Corfu populations of these species were investigated by an integrative approach including analysis of morphological features of shell and distal genitalia as well as molecular features of selected mitochondrial and nuclear gene fragments to establish the relationships between Corfu *M. claustralis* and *M. cartusiana* as well as between Corfu and Italian *M. parumcincta*. Shell features did not differentiate the pairs analysed, i.e. *Monacha claustralis* vs *M. cartusiana* and Corfu vs Italian *M. parumcincta*, whereas features of distal genitalia structure and nucleotide sequences of mitochondrial genes (COI and 16SrDNA) distinguished them significantly. Nuclear gene sequences (ITS2 flanked with 5.8S and 28SrDNA fragments) also differentiated between Corfu and Italian *M. parumcincta*. It is therefore postulated that these two pairs are composed of four separate species: *M. claustralis*, *M. cartusiana*, Corfu *M. parumcincta*, and Italian *M. parumcincta*, which are distinct from each other and from the other species of the genus *Monacha* used here for comparison (the six lineages of *M. cantiana* s.l. and *M. pantanellii*).

## ﻿Introduction

Among the hygromiids, *Monacha* Fitzinger, 1833 is the most speciose genus, including almost a hundred species (93 according to MolluscaBase 2024), widespread from Britain and north-western France to the Caucasus, Middle East, and north African coast (Hausdorf 2000a, 2000b; Welter-Schultes 2012; Neiber and Hausdorf 2017 and other references therein).

The phylogeny and biogeography of the genus were addressed by Neiber and Hausdorf (2017) on the basis of anatomical features (reproductive system) and molecular data (mitochondrial and nuclear gene sequences). Since publication of their paper, eight subgenera have been accepted: *Aegaeotheba* Neiber & Hausdorf, 2017, *Metatheba* Hesse, 1914, *Monacha* s.s., *Paratheba* Hesse, 1914, *Platytheba* Pilsbry, 1894, *Pontotheba* Neiber & Hausdorf, 2017, *Rhytidotheba* Neiber & Hausdorf, 2017 and *Trichotheba* Neiber & Hausdorf, 2017. Most are restricted to Anatolia and the Caucasus, regarded as the area of origin of the genus; others colonised southern Europe, the Crimean Peninsula, and the Middle East (Neiber and Hausdorf 2017).

Species level taxonomy is still in progress. To date only a few *Monacha* species have been studied using an integrative approach, with thorough investigation of the geographical structure of their morphological and molecular variations (e.g. Pieńkowska et al. 2018b, 2019, 2024; Williams et al. 2024). Moreover, many early established taxa have taxonomic and nomenclatural issues requiring clarification. This is also true of species living in countries that have been studied intensively from a malacological point of view, such as those of southern Europe.

Here we addressed the taxonomic nomenclatural revision of the *Monacha* species occurring on Corfu (Kérkyra, Ionian islands, NW Greece), because this island is the type locality of two early-established species of the genus: *Monacha claustralis* (Rossmässler, 1834) and *Monacha parumcincta* (Rossmässler, 1834) (Forcart 1965; Welter-Schultes 2012). The former is probably native to the Balkan Peninsula and western Turkey but its range has now expanded into central and eastern Europe as far as Germany, Poland, Ukraine, and Georgia (Hausdorf 2000a; Pieńkowska et al. 2015, 2018a; Hutchinson et al. 2019; Gural-Sverlova and Gural 2022). Conchologically, it is very similar to *Monacha cartusiana* (Müller, 1774) from which it is distinguished by some anatomical features and molecular sequences (Hausdorf 2000a; Pieńkowska et al. 2015, 2018a; Neiber and Hausdorf 2017). However, specimens of uncertain attribution – due to intermediate/divergent anatomical and molecular traits – were recently discovered in the non-native range, raising doubts about the distinctness and reproductive isolation of the two species (Čejka et al. 2020; Gural-Sverlova and Gural 2022, 2023; Lesicki et al. 2024; Williams et al. 2024). The second Corfu species – *M. parumcincta* – is reported from central and southern Italy and the Balkan Peninsula (Welter-Schultes 2012; Bank and Neubert 2017). Unfortunately no one has ever had the opportunity to study the Balkan populations so their conspecificity with Italian populations is uncertain (Welter-Schultes 2012; Pieńkowska et al. 2018b).

In order to settle their relationships, a new study of the two species was conducted on new populations from their type locality (Corfu) using an integrative approach which included morphological (shell and anatomy) and molecular (mitochondrial and nuclear gene sequences) data. The aims of this research were to study the relationships between topotypical *Monacha claustralis* and *Monacha cartusiana* and between topotypical *Monacha parumcincta* and the Italian populations so far assigned to this species. The results of the study allowed us to redefine the Corfu and some other related species.

## ﻿Materials and methods

### ﻿Taxonomic samples

**Figure 1.**
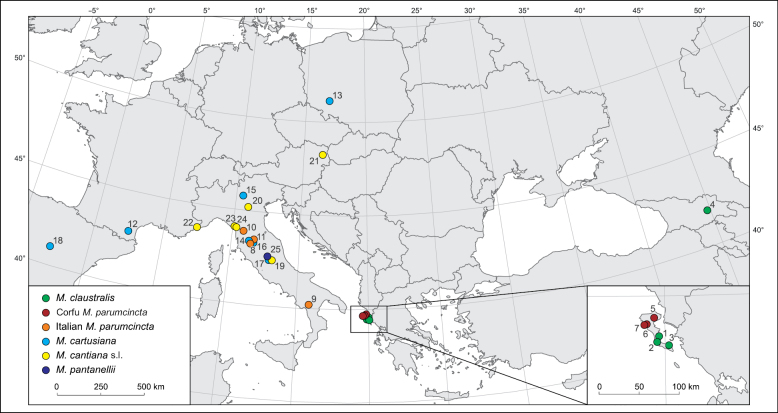
Map of localities of the populations of *Monacha claustralis* (1–3) and *M. parumcincta* (5–7) on Corfu island together with localities of Italian *M. parumcincta* (8–11) and the other species compared in this paper (*M. claustralis* 4, *M. cartusiana* 12–18, *M. cantiana* s.l. 19–24 and *M. pantanellii* 25) (for details see Table 1).

Three populations each of *Monacha claustralis* and *Monacha parumcincta* from Corfu (Fig. 1, Table 1) were considered for an analysis of their morphological (shell and genitalia) and molecular features with the aim of establishing the taxonomic identity of these species. These populations from Corfu were compared morphologically and molecularly with those of *Monacha cartusiana* from Italy (three populations) and *Monacha parumcincta* from Italy (four populations). As well, representatives of *Monacha cartusiana* from France (one population), Spain (one population), and Poland (one population), *Monacha claustralis* from Georgia (one population), *Monacha cantiana* s.l. (Montagu, 1803) (six populations from Italy, Austria, and France), and *Monacha pantanellii* (De Stefani, 1879) from Italy (one population) were used in comparative molecular studies (Table 1). Sequences from these specimens were deposited in GenBank during previous studies (Neiber and Hausdorf 2017; Pieńkowska et al. 2018a, 2018b, 2019, 2020, 2022, 2024) and several new sequences of mitochondrial (16SrDNA) and nuclear genes (ITS2 flanked with 5.8SrDNA and 28SrDNA) (Table 1) were also used in molecular analysis. Sequences of *Trochulus hispidus* (Linnaeus, 1758) from GenBank (Neiber and Hausdorf 2015, 2017; Neiber et al. 2017; Caro et al. 2019) were used as an outgroup to construct phylogenetic trees.

**Table 1.** List of localities of populations of Corfu *Monacha claustralis* and *M. parumcincta* used for molecular and morphological (SH shell, AN genitalia) analysis. Populations of other *Monacha* species used in comparative molecular and anatomical research are also listed.

Localities				Acronym for population	Current taxonomy	Designation of voucher specimens	COI		Long 16SrDNA		5.8S rDNA + ITS2 + 28S rDNA		PCA and RDA	Figs
No.	Coordinates	Country and site	Collector / no. of specimens / collection				New haplotype	GenBank number	New haplotype	GenBank number	New haplotype	GenBank number		
1	39°32'29.98"N, 19°54'50.49"E	Greece, Corfu [Kérkyra], Benitses, 5–10 m asl	A. Benocci, G. Manganelli and L. Manganelli / 24.10.2022 / 9 / FGC 52237	CLA BEN	*M. claustralis*	Ben1	COI 1	PP947873	16S 1	PP949387	ITS2 1	PP947951	SH – AN	10 (AN)
						Ben2	COI 1	PP947874	16S 2	PP949388	ITS2 2	PP947952		
						Ben3	COI 2	PP947875	16S 3	PP949389	ITS2 3	PP947953		
						Ben4	COI 3	PP947876	16S 2	PP949390	ITS2 4	PP947954		
						Ben5	COI 3	PP947877	16S 2	PP949391	ITS2 5	PP947955		
						Ben6	COI 3	PP947878	16S 2	PP949392	ITS2 3	PP947956		
2	39°28'36.32"N, 19°53'06.07"E	Greece, Corfu [Kérkyra], Gardiki, 40 m asl	A. Benocci, G. Manganelli and L. Manganelli / 26.10.2022 / 5 / FGC 52297	CLA GAR	*M. claustralis*	Gar6	COI 4	PP947879	16S 4	PP949393	ITS2 4	PP947957	SH – AN	2 (SH)
						Gar7	COI 5	PP947880	16S 5	PP949394	ITS2 4	PP947958		9 (AN)
						Gar8	COI 6	PP947881	16S 6	PP949395	ITS2 4	PP947959		
						Gar9	COI 7	PP947882	16S 7	PP949396	ITS2 4	PP947960		
						Gar10	COI 8	PP947883	16S 4	PP949397	ITS2 4	PP947961		
3	39°26'14.67"N, 20° 03'19.77"E	Greece, Corfu [Kérkyra], Molos cemetery, 10 m asl	A. Benocci, G. Manganelli and L. Manganelli / 26.10.2022 / 6 / FGC 52301	CLA MOL	*M. claustralis*	Mol6	COI 1	PP947884	16S 8	PP949398	ITS2 4	PP947962	SH – AN	2 (SH)
						Mol7	COI 1	PP947885	16S 8	PP949399	ITS2 4	PP947963		8 (AN)
						Mol8	COI 9	PP947886	16S 9	PP949400	ITS2 4	PP947964		
						Mol9	COI 3	PP947887	16S 8	PP949401	ITS2 4	PP947965		
						Mol10	COI 3	PP947888	16S 8	PP949402	ITS2 4	PP947966		
						Mol11	COI 3	PP947889	16S 8	PP949403	ITS2 4	PP947967		
4	41°53'43"N, 44°46'09"E	Georgia, Mtskheta-Mtianeti, SE of Saguramo, between village and cemetery, 650 m asl	Hausdorf and Neiber (2017) / 1 / ZMH 86012 (1775)		*M. claustralis*			KX507199		KX495388		KX495441		
5	39°45'07.70"N, 19°50'42.98"E	Greece, Corfu [Kérkyra], Pantokrator, 900 m asl	A. Benocci, G. Manganelli and L. Manganelli / 24.10.2022 / 5 / FGC 52284	PAR-K PNK1	Corfu *M. parumcincta*	Pnk1-1	COI 10	PP947890	16S 10	PP949404	ITS2 6	PP947968	SH – AN	5 (SH)
						Pnk1-2	COI 11	PP947891	16S 11	PP949405	ITS2 7	PP947969		15 (AN)
						Pnk1-3	COI 11	PP947892	16S 12	PP949406	ITS2 6	PP947970		
						Pnk1-4	COI 11	PP947893	16S 11	PP949407	ITS2 6	PP947971		
						Pnk1-5	COI 11	PP947894	16S 11	PP949408	ITS2 6	PP947972		
6	39°40'49.64"N, 19°44'12.86"E	Greece, Corfu [Kérkyra], Paleokastritsa, Bimbos supermarket, 90 m asl	A. Benocci, G. Manganelli and L. Manganelli / 25.10.2022 / 8 / FGC 52306	PAR-K BIM1	Corfu *M. parumcincta*	Bim1-1	COI 12	PP947895	16S 13	PP949409	ITS2 8	PP947973	SH – AN	5 (SH)
						Bim1-2	COI 13	PP947896	16S 14	PP949410	ITS2 8	PP947974		14 (AN)
						Bim1-3	COI 14	PP947897	16S 15	PP949411	ITS2 8	PP947975		
						Bim1-4	COI 13	PP947898	16S 16	PP949412	ITS2 8	PP947976		
						Bim1-5	COI 13	PP947899	16S 17	PP949413	ITS2 8	PP947977		
7	39°40'17.43"N, 19°42'04.51"E	Greece, Corfu [Kérkyra], Paleokastritsa, along the road to monastery of Paleokastritsa, 10-30 m asl	A. Benocci, G. Manganelli and L. Manganelli / 25.10.2022 / 16 / FGC 52333	PAR-K PAL	Corfu *M. parumcincta*	Pale1	COI 15	PP947900	16S 18	PP949414	ITS2 8	PP947978	SH – AN	5 (SH)
						Pale2	COI 16	PP947901	16S 19	PP949415	ITS2 8	PP947979		13 (AN)
						Pale3	COI 17	PP947902	16S 20	PP949416	ITS2 8	PP947980		
						Pale4	COI 13	PP947903	16S 21	PP949417	ITS2 8	PP947981		
						Pale5	COI 17	PP947904	16S 20	PP949418	ITS2 8	PP947982		
						Pale6	COI 17	PP947905	16S 20	PP949419	ITS2 8	PP947983		
8	43°18'59.40"N, 11°30'04.20"E	Italy, Tuscany, La Casella (Asciano, Siena), 275 m asl	G. Manganelli / 04.10.2015 / 11 / FGC 44077	PAR-I	Italian *M. parumcincta*	Cas-1		MG208959	16S 22	PP949420	ITS2 9	PP947984	SH – AN	6 (SH) 16 (AN)
9	40°13'25.49"N, 15°52'17.07"E	Italy, Basilicata, along the road from Moliterno to Fontana d’Eboli (Moliterno, Potenza)	A. Hallgass / 10.2012 / 5 / FGC 42962	PAR-I	Italian *M. parumcincta*	15FG-1		MG208944	16S 23	PP949421	ITS2 10	PP947985	SH – AN	6 (SH) 16 (AN)
						15FG-2		MG208947	16S 24	PP949422	ITS2 11	PP947986		
10	43°54'18.00"N, 10°49'13.63"E	Italy, Tuscany, Chiesina, Nievole (Montecatini Terme, Pistoia), 60 m asl	A. Hallgass / 20.10.2013 / 2 / FGC 41562	PAR-I	Italian *M. parumcincta*	Nie-2		MG208949	16S 25	PP949423				
11	43°30'19.55"N, 11°38'54.92"E	Italy, Tuscany, A1 highway, rest area Romita est (Pergine Valdarno, Arezzo), 60 m asl	A. Hallgass / 10.2013 / 6 / FGC 41561	PAR-I	Italian *M. parumcincta*	Are-5		MG208950	16S 26	PP949424	ITS2 9	PP947987	SH – AN	6 (SH)
						Are-1		MG208956	16S 26	PP949425	ITS2 9	PP947988		
12	42°52'09.7"N, 02°29'06.0"E	France, Occitania, Aude, Cubières-sur-Cinoble, roadside, 419 m asl	M. Proćków / 28.06.2018 / 5 / DCBC & MNHW-F.18.38	CUR	*M. cartusiana*	Cur2		ON332653		ON350961		ON332790		
						Cur4		ON332655		ON350963	ITS2 12	PP947989		
						Cur5	COI 18	PP947906		ON350964	ITS2 13	PP947990		
13	51°08'30.5"N, 16°56'55.9"E	Poland, Wrocław-Pilczyce (Mączna St.), 80 m E of Ślęza River bank, 120 m asl	E. Kowalska / 8.06.2023 / 5 / and 17.09.2023 / 10 / DCBC	WRO	*M. cartusiana*	Wro10	COI 19	PP947907	16S 27	PP949426	ITS2 14	PP947991		
						Wro11	COI 19	PP947908	16S 27	PP949427	ITS2 14	PP947992		
						Wro12	COI 19	PP947909	16S 27	PP949428	ITS2 14	PP947993		
						Wro13	COI 20	PP947910	16S 28	PP949429	ITS2 14	PP947994		
						Wro14	COI 19	PP947911	16S 27	PP949430	ITS2 14	PP947995		
						Wro15	COI 20	PP947912	16S 29	PP949431	ITS2 14	PP947996		
						Wro20	COI 20	PP947913	16S 27	PP949432	ITS2 14	PP947997		
						Wro22	COI 21	PP947914	16S 27	PP949433	ITS2 14	PP947998		
						Wro23	COI 21	PP947915	16S 27	PP949434	ITS2 14	PP947999		
						Wro24	COI 19	PP947916	16S 27	PP949435	ITS2 14	PP948000		
						Wro16	COI 22	PP947917	16S 30	PP949436	ITS2 14	PP948001		
						Wro21	COI 22	PP947918	16S 38	PP949437	ITS2 14	PP948002		
14	43°24'34"N, 11°17'22"E	Italy, Tuscany, Quattrovie near Quercegrossa (Siena), 305 m asl	G. Manganelli / 20.06.2023 / 5 / FGC 54942 G. Manganelli / 24.09.2023 / 6 / FGC 55672		*M. cartusiana*	Que1	COI 23	PP947919	16S 31	PP949438	ITS2 1	PP948003	SH – AN	3 (SH)
						Que2	COI 24	PP947920	16S 32	PP949439	ITS2 1	PP948004		11 (AN)
						Que3	COI 23	PP947921	16S 31	PP949440	ITS2 1	PP948005		
						Que4	COI 24	PP947922	16S 31	PP949441	ITS2 1	PP948006		
						Que5	COI 25	PP947923	16S 33	PP949442	ITS2 1	PP948007		
15	45°46'38"N, 10°30'12"E	Italy, Lombardy, Anfo towards Ponte Caffaro, calcareous rocks at branch towards Tre Casali, 400 m asl	Hausdorf and Neiber (2017) / 1 / ZMH 51710 (1594)		*M. cartusiana*			KX507189		KX495378		KX495431		
16	43°18.45'N, 11°28.88'E	Italy, Tuscany, Stazione di Castelnuovo Berardenga (Asciano, Siena), 210 m asl	G. Manganelli / 01.11.1981 / 5 / FGC 3430		*M. cartusiana*								SH – AN	3 (SH) 11 (AN)
17	? 42°28.85'N, 12°50.84'E	Italy, Latium, Lago Lungo (Rieti), 370 m asl	F. Giusti / 14.08.1966 / 5 / FGC 23875		*M. cartusiana*								SH - AN	
18	41°00'00"N, 02°38'00"W	Spain, Castilla-La Mancha, Cañon del Río Dulce	Hausdorf and Neiber (2017) / 1 / SP166		*M. cantiana* s.str.			KX507235		KX495429		KX495479		
19	42°28'41.05"N, 13°05'09.46"E	Italy, Latium, Gole del Velino, near Sigillo (Posta, Rieti), 594 m asl	A. Hallgass / 30.09.2012 / 8 / FGC 42960	CAN-1	*M. cantiana* s.s.			MG208905		OR918428		OR917402		
								MG208910		OR918429		OR917403		
20	45°11'59.85"N, 10°58'49.30"E	Italy, Venetum, Sorgà (Verona), 22 m asl	A. Hallgass / 09.2012 / 6 / FGC 42964	CAN-2	*M. cantiana* s.l.			MG208925		OR918435		OR917406		
								MG208928		OR918436		OR917407		
21	48°15'25.50"N, 16°30'46.38"E	Austria, Breitenlee, abandoned railway station	M. Duda / 09.2015 / 3 / FGC 44020	CAN-3	*M. cantiana* s.l.			MG208938		OR918437		OR917408		
22	43°46'11.79"N, 07°22'21.50"E	France, Alpes-Maritimes, Vallée de Peillon, Sainte Thècle	A. Hallgass / 24.10.2011/ 5 / FGC 40320	CAN-4	*M. cantiana* s.l. (*M. cemenelea*)			MG208939		OR918438		OR917409		
								MG208940		OR918439		OR917410		
23	44°05'56.8"N, 10°07'08.5"E	Italy, Tuscany, Apuan Alps, Piastra (Carrara, Massa Carrara), 290 m asl	A. Hallgass / 13.10.2013 / 5 / FGC 41563	CAN-5	*M. cantiana* s.l.			MK066938	16S 34	PP949443	ITS2 15	PP948008		
24	44°03'25.5"N, 10°16'01.0"E	Italy, Tuscany, Apuan Alps, 1 km E of Campagrina (Stazzema, Lucca), 769 m asl	A. Hallgass / 22.10.2011 / 5 / FGC 40322	CAN-6	*M. cantiana* s.l.			MK066944	16S 35	PP949444	ITS2 16	PP948009		
								MK066943	16S 36	PP949445	ITS2 16	PP948010		
25	42°40'03.0"N, 12°44'31.8"E	Italy, Umbria, Monte Fionchi, 900 m NE of Torrecola (Spoleto, Perugia), 680 m asl	A. Hallgass / 2010 / 5 / FGC 38944	FIO	*M. pantanellii*			MT380015	16S 37	PP949446	ITS2 17	PP948011		

### ﻿Material examined

The material examined by an integrative approach based on morphological (shell and genitalia) and molecular analysis is listed in Table 1; other material already published used for comparison has been described in previous papers (Pieńkowska et al. 2018a, 2018b, 2019, 2020, 2022, 2024: Table 1). The following data is provided for each population: geographic coordinates, country and region, short description of collection site, name of collector/s, date, number of specimens studied, and the depository where they are stored.

### ﻿Morphological study

Fifty-three specimens of the four lineages (Table 1: *M. claustralis*, *M. cartusiana*, Corfu *M. parumcincta*, and Italian *M. parumcincta*) were considered for shell variability. Seven shell variables were measured to the nearest 0.1 mm using ADOBE PHOTOSHOP 7.0.1 on digital images of standard apertural and umbilical views taken with a Canon EF 100 mm 1:2.8 L IS USM macro lens mounted on a Canon F6 camera (see also Pieńkowska et al. 2018b: fig. 1): **AH** aperture height, **AW** aperture width, **LWaH** height of adapical sector of last whorl, **LWmH** height of medial sector of last whorl, **SD** shell diameter, **SH** shell height, **UD** umbilicus diameter.

Sixty specimens of the four lineages (Table 1: *M. claustralis*, *M. cartusiana*, Corfu *M. parumcincta*, and Italian *M. parumcincta*) were analysed for anatomical variability. Snail bodies were dissected under a light microscope (Wild M5A or Zeiss SteREO Lumar V12). Anatomical details were drawn using a Wild camera lucida, and labelled with the following acronyms (see also Pieńkowska et al. 2018b: fig. 2): **BC** bursa copulatrix, **BW** body wall, **DBC** duct of bursa copulatrix, **DG** digitiform glands, **E** epiphallus (from base of flagellum to beginning of penial sheath), **F** flagellum, **FO** free oviduct, **GA** genital atrium, **GAR** genital atrium retractor, **P** penis, **PP** penial papilla, **PSO** prostatic section of spermoviduct, **SOD** spermoviduct, **USO** uterine section of spermoviduct, **V** vagina, **VA** vaginal appendix (also known as appendicula), **VD** vas deferens, **VR** vaginal refringent ring, **VS** vaginal sac.

**Figure 2.**
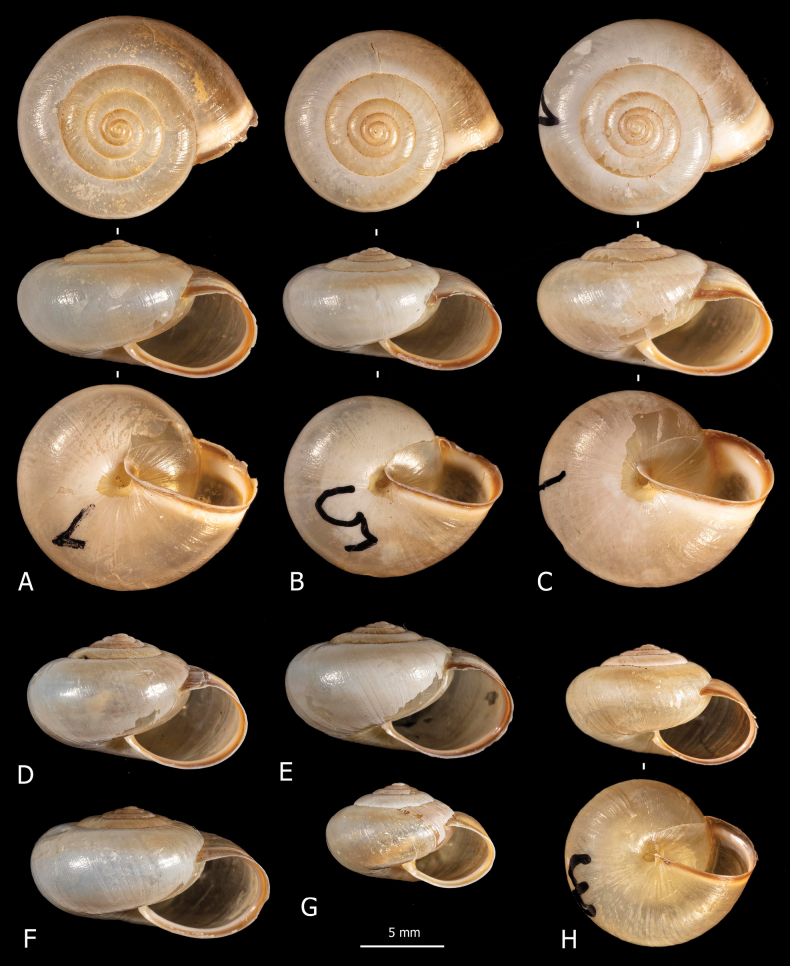
Shells of *Monacha claustralis* from Corfu. Specimens from Molos cemetery, A. Benocci, G. Manganelli and L. Manganelli leg. 26.10.2022 (FGC 52301) (A, B, D, F) and Gardiki, A. Benocci, G. Manganelli and L. Manganelli leg. 26.10.2022 (FGC 52297) (C, E, G, H).

Six anatomical variables (DBC, E, F, P, V, VA) were measured with callipers under a light microscope (0.01 mm) (Pieńkowska et al. 2018b: fig. 2). Multivariate ratio analysis (MRA; Baur and Leuenberger 2011) was performed on the shell and genital data. The method is specifically designed to interpret results from principal component analysis (PCA) and linear discriminant analysis (LDA) in terms of body ratios that can be used for taxonomic inference. This approach is particularly suited for distinguishing size and shape components, a critical aspect in many morphometric studies.

Principal component analysis was performed in MRA shape space. The principal components (PCs) were interpreted using the PCA Ratio Spectrum, a graphic tool that identifies the ratios most strongly associated with each shape component, i.e. ratios lying close to opposite ends of the spectrum (Baur and Leuenberger 2011; Baur et al. 2014). To investigate potential allometric effects – variations in shape linked to size – isosize (calculated as the geometric mean of the original measurements) was plotted against significant PCs. The Allometry Ratio Spectrum was also used to determine which variables showed the greatest size-related influence. The LDA ratio extractor was then employed to identify the body ratios that best discriminated taxa. Standard distance (Dij) and the delta (δ) measurements were used to quantify the relative contributions of size and shape to group differentiation. Data analysis was performed using Rstudio (R v. 4.2.1; R Core Team 2021), along with scripts provided by Baur and Leuenberger (2020).

### ﻿Molecular study

Thirty-three specimens from six Corfu *Monacha* populations (Table 1: *M. claustralis*, Corfu *M. parumcincta*) were used for molecular analysis. New sequences obtained from six specimens of Italian *M. parumcincta* populations, two specimens of French, twelve of Polish and five of Italian *M. cartusiana* populations, three specimens of *M. cantiana* s.l. populations, as well as one specimen of *M. pantanellii* (Table 1) were also compared with sequences deposited for these species in GenBank (Neiber and Hausdorf 2017, Pieńkowska et al. 2018a, 2018b, 2019, 2020, 2022, 2024). Molecular methods including DNA extraction, amplification, and sequencing are described in our previous papers (Pieńkowska et al. 2018a, 2018b).

Two mitochondrial and three nuclear gene fragments were analysed, namely cytochrome c oxidase subunit 1 (COI), 16S ribosomal DNA (16SrDNA), and an internal transcribed spacer 2 of rDNA (ITS2) flanked by the 3’end of 5.8SrDNA and the 5’end of 28SrDNA, respectively. Sequences were edited by eye using BioEdit, v. 7.0.6 (Hall 1999; BioEdit 2017) and aligned using ClustalW, implemented in BioEdit (Thompson et al. 1994). Fragments of COI were amplified using two pairs of primers: F01/R04 (Dabert et al. 2010) or LCO1490/HCO2198 (Folmer et al. 1994) and aligned according to the translated amino acid sequences. Fragments of 16SrDNA were amplified using 16Scs1/16Scs2 primers (Chiba 1999). Sequences containing the 3’end of 5.8SrDNA, complete sequence of ITS2 and 5’end of 28S rDNA were amplified using a pair of primers: LSU1/LSU3 (Wade and Mordan 2000). The ends of all sequences were trimmed. After trimming, the lengths of sequences were 615 bp for COI, 871 bp for 16SrDNA, and 748–755 bp for ITS2 flanked by the 3’end of 5.8SrDNA and the 5’end of 28SrDNA (45 bp 5.8SrDNA + 488–495 bp ITS2 + 215 bp 28SrDNA). The borders of the ITS2 sequence were searched for using ITS2-Database (http://its2.bioapps.biozentrum.uni-wuerzburg.de) (Eddy 1998; Koetschan et al. 2010). The sequences were collapsed to haplotypes using the programme ALTER (Alignment Transformation EnviRonment) (Glez-Peña et al. 2010). The following alignments were made for phylogenetic inference: 615 bp long for COI, 871 positions long for 16SrDNA, 778 positions long for ITS2 flanked by the 3’end of 5.8SrDNA and the 5’end of 28SrDNA. Finally, the sequences of COI, 16SrDNA and ITS2 were concatenated (Table 2). Two sets of concatenated sequences were created: 1) COI16S 1486 positions in length (615 COI + 871 16SrDNA); 2) CS 2264 positions in length (615 COI + 871 16SrDNA + 778 ITS2 with flanks).

**Table 2.** Concatenated sequences of COI+16SrDNA (COI16S) and COI+16SrDNA+ITS2 (CS) used in MEGA7/IQ-Tree/BI analysis (Figs 19, 21, respectively).

Concatenated sequence	COI haplotype	16SrDNA haplotype	Concatenated sequence	COI haplotype	16SrDNA haplotype	ITS2 haplotype	Locality (population No.: specimen No.) *
*Monacha claustralis* (Greece: Corfu)							
COI16S 1	1	1	CS 1	1	1	1	Greece: Corfu [Kérkyra], Benitses (1: Ben1)
COI16S 2	1	2	CS 2	1	2	2	Greece: Corfu [Kérkyra], Benitses (1: Ben2)
COI16S 3	2	3	CS 3	2	3	3	Greece: Corfu [Kérkyra], Benitses (1: Ben3)
COI16S 4	3	2					Greece: Corfu [Kérkyra], Benitses (1: Ben4, Ben5, Ben6)
			CS 4	3	2	4	Greece: Corfu [Kérkyra], Benitses (1: Ben4)
			CS 5	3	2	5	Greece: Corfu [Kérkyra], Benitses (1: Ben5)
			CS 6	3	2	3	Greece: Corfu [Kérkyra], Benitses (1: Ben6)
COI16S 5	4	4	CS 7	4	4	4	Greece: Corfu [Kérkyra], Gardiki (2: Gar6)
COI16S 6	5	5	CS 8	5	5	4	Greece: Corfu [Kérkyra], Gardiki (2: Gar7)
COI16S 7	6	6	CS 9	6	6	4	Greece: Corfu [Kérkyra], Gardiki (2: Gar8)
COI16S 8	7	7	CS 10	7	7	6	Greece: Corfu [Kérkyra], Gardiki (2: Gar9)
COI16S 9	8	4	CS 11	8	4	4	Greece: Corfu [Kérkyra], Gardiki (2: Gar10)
COI16S 10	1	8	CS 12	1	8	4	Greece: Corfu [Kérkyra], Molos cemetery (3: Mol6, Mol7)
COI16S 11	9	9	CS 13	9	9	4	Greece: Corfu [Kérkyra], Molos cemetery (3: Mol8)
COI16S 12	3	8	CS 14	3	8	6	Greece: Corfu [Kérkyra], Molos cemetery (3: Mol9, Mol10, Mol11)
*Monacha claustralis* ? (*Monacha cartusiana*) (Poland: Wrocław)							
COI16S 13	22	30	CS 15	21	30	14	Poland: Wrocław, Pilczyce (13: Wro16)
COI16S 14	22	38	CS 16	21	38	14	Poland: Wrocław, Pilczyce (13: Wro21)
*Monacha claustralis* (Georgia: Mtskheta-Mtianeti)							
	KX507199	KX495388		KX507199	KX495388	KX495441	Georgia: Mtskheta-Mtianeti, SE of Saguramo (4, Neiber and Hausdorf 2017: ZMH 86012)
*Monacha parumcincta* (Greece: Corfu)							
COI16S 15	10	10	CS 17	10	10	7	Greece: Corfu [Kérkyra], Pantokrator (5: Pnk1-1)
COI16S 16	11	11	CS 18	11	11	8	Greece: Corfu [Kérkyra], Pantokrator (5: Pnk1-2)
			CS 19	11	11	7	Greece: Corfu [Kérkyra], Pantokrator (5: Pnk1-4, Pnk1-5)
COI16S 17	11	12	CS 20	11	12	7	Greece: Corfu [Kérkyra], Pantokrator (5: Pnk1-3)
COI16S 18	12	13	CS 21	12	13	9	Greece: Corfu [Kérkyra], Paleokastritsa, Bimbos (6: Bim1-1)
COI16S 19	13	14	CS 22	13	14	9	Greece: Corfu [Kérkyra], Paleokastritsa, Bimbos (6: Bim1-2)
COI16S 20	14	15	CS 23	14	15	9	Greece: Corfu [Kérkyra], Paleokastritsa, Bimbos (6: Bim1-3)
COI16S 21	13	16	CS 24	13	16	9	Greece: Corfu [Kérkyra], Paleokastritsa, Bimbos (6: Bim1-4)
COI16S 22	13	17	CS 25	13	17	9	Greece: Corfu [Kérkyra], Paleokastritsa, Bimbos (6: Bim1-5)
COI16S 23	15	18	CS 26	15	18	9	Greece: Corfu [Kérkyra], Paleokastritsa, monastery (7: Pale1)
COI16S 24	16	19	CS 27	16	19	9	Greece: Corfu [Kérkyra], Paleokastritsa, monastery (7: Pale2)
COI16S 25	17	20	CS 28	17	20	9	Greece: Corfu [Kérkyra], Paleokastritsa, monastery (7: Pale3, Pale5, Pale6)
COI16S 26	13	21	CS 29	13	21	9	Greece: Corfu [Kérkyra], Paleokastritsa, monastery (7: Pale4)
*Monacha parumcincta* (Italy)							
COI16S 27	MG208959	22	CS 30	MG208959	22	10	Italy: Tuscany, La Casella (Asciano, Siena) (8: Cas1)
COI16S 28	MG208944	23	CS 31	MG208944	23	9	Italy, Basilicata, Moliterno (9: 15FG-1)
COI16S 29	MG208947	24	CS 32	MG208947	24	10	Italy, Basilicata, Moliterno (9: 15FG-2)
COI16S 30	MG208949	25					Italy, Tuscany, Nievole (910: Nie-2)
COI16S 31	MG208956	26	CS 33	MG208956	26	9	Italy, Tuscany, Arezzo (11: Are-1)
COI16S 32	MG208950	26	CS 34	MG208950	26	9	Italy, Tuscany, Arezzo (11: Are-5)
*Monacha cartusiana* (France)							
	ON332653	ON350961	CS 35	ON332653	ON350961	ON332790	France, Occitania, Aude, Cubières-sur-Cinoble (12: Cur2)
	ON332655	ON350963	CS 36	ON332655	ON350963	12	France, Occitania, Aude, Cubières-sur-Cinoble (12: Cur4)
COI16S 33	18	ON350964	CS 37	18	ON350964	13	France, Occitania, Aude, Cubières-sur-Cinoble (12: Cur5)
*Monacha cartusiana* (Poland)							
COI16S 34	19	27	CS 38	19	27	14	Poland, Wrocław-Pilczyce (13: Wro10, Wro11, Wro12, Wro14, Wro24)
COI16S 35	20	27	CS 39	20	27	14	Poland, Wrocław-Pilczyce (13: Wro20)
COI16S 36	20	28	CS 40	20	28	14	Poland, Wrocław-Pilczyce (13: Wro13)
COI16S 37	20	29	CS 41	20	29	14	Poland, Wrocław-Pilczyce (13: Wro15)
COI16S 38	21	27	CS 42	21	27	14	Poland, Wrocław-Pilczyce (13: Wro22, Wro23)
*Monacha cartusiana* (Italy)							
COI16S 39	23	31	CS 43	23	31	1	Italy, Tuscany, Quattrovie (14: Que1, Que3, Que4)
COI16S 40	24	32	CS 44	24	32	1	Italy, Tuscany, Quattrovie (14: Que2)
COI16S 41	25	33	CS 45	25	33	1	Italy, Tuscany, Quattrovie (14: Que5)
	KX507189	KX495378		KX507189	KX495378	KX495431	Italy, Lombardy, Anfo towards Ponte Caffaro (15; Neiber and Hausdorf 2017: ZMH 51710, 1594)
*Monacha cartusiana* (Spain)							
	KX507235	KX495429		KX507235	KX495429	KX495479	Spain, Castilla-La Mancha, Cañon del Río Dulce (18; Neiber and Hausdorf 2017: SP166)
*Monacha cantiana* CAN-1							
	MG208905	OR918428		MG208905	OR918428	OR917402	Italy: Latium, Gole del Velino (19: 4FG-1)
	MG208910	OR918429		MG208910	OR918429	OR917403	Italy: Latium, Gole del Velino (19: 4FG-2)
*Monacha cantiana* s.l. CAN-2							
	MG208925	OR918435		MG208925	OR918435	OR917406	Italy: Venetum, Sorgà (20: 12FG-1)
	MG208928	OR918436		MG208928	OR918436	OR917407	Italy: Venetum, Sorgà (20: 12FG-2)
*Monacha cantiana* s.l. CAN-3							
	MG208938	OR918437		MG208938	OR918437	OR917408	Austria: Breitenlee (21: Dud2)
*Monacha cantiana* s.l. CAN-4 (*Monacha cemenelea*)							
	MG208939	OR918438		MG208939	OR918438	OR917409	France: Alpes-Maritimes, Sainte Thècle (22: 3FG-1)
	MG208940	OR918439		MG208940	OR918439	OR917410	France: Alpes-Maritimes, Sainte Thècle (22: 3FG-2)
*Monacha cantiana* s.l. CAN-5							
COI16S 42	MK066938	34	CS 46	MK066938	34	15	Italy: Apuan Alps, Piastra (23: Pia2)
*Monacha cantiana* s.l. CAN-6							
COI16S 43	MK066944	35	CS 47	MK066944	35	16	Italy: Tuscany, Apuan Alps, Campagrina (24: 5FG-1)
COI16S 44	MK066943	36	CS 48	MK066943	36	16	Italy: Tuscany, Apuan Alps, Campagrina (24: 5FG-2)
*Monacha pantanellii*							
COI16S 45	MT380015	37	CS 49	MT380015	37	17	Italy: Umbria, Monte Fionchi (25: Fio3)
*Trochulus hispidus*							
	KX507209	KX495398		KX507209	KX495398	KX495451	Germany: Hamburg (Neiber and Hausdorf 2017: ZMH 119338, 2410)

Estimates of genetic distances between the COI sequences obtained in this study and other sequences from GenBank were conducted with MEGA7 using the Kimura two-parameter model (K2P) (Kimura 1980; Kumar et al. 2016). All positions with gaps and missing data were eliminated. There was a total of 615 positions in the final dataset. The analysis involved 50 nucleotide sequences.

To infer phylogenetic relationships, the following software programs were used: MEGA7 (Hasegawa et al. 1985; Nei and Kumar 2000; Kumar et al. 2016), IQ-Tree (Nguyen et al. 2015), and MrBayes 3.2.6 (Ronquist et al. 2012).

For each alignment file, best nucleotide substitution models were specified according to the Bayesian Information Criterion (BIC) by means of MEGA7 software: HKY+G+I for analysis of COI, T92+G+I of concatenated sequences COI+16SrDNA, GTR+G of 16SrDNA, GTR+G+I of concatenated sequences COI+16SrDNA+ITS2 (with 5.8S and 28SrDNA) and T92 for analysis of ITS2 (with flanks) (Jukes and Cantor 1969; Hasegawa et al. 1985; Tamura 1992; Kumar et al. 2016). Best substitution models were inferred according to BIC for each of the partitions by MODELFINDER (Kalyaanamoorthy et al. 2017) implemented in IQ-Tree: HKY+F+I+G4 (Hasegawa et al. 1985) for analysis of COI, GTR+F+I+G4 (Tavaré 1986) of 16SrDNA, and K2P+G4 (Kimura 1980) of ITS2 (flanked by 5.8SrDNA and 28SrDNA). Phylogenetic analysis performed with IQ-Tree for two sets of concatenated sequences (see above: COI16S and CS) was done dividing the data set into two or three partitions (Chernomor et al. 2016): 1) COI, 2) 16SrDNA or 1) COI, 2) 16SrDNA, 3) 5.8SrDNA+ ITS2 + 28SrDNA with best substitution models: HKY+F+I+G4 for partition 1, GTR+F+I+G4 for partition 2, and K2P+I+G4 for partition 3. Bayesian analysis of concatenated sequences COI+16SrDNA and COI+16SrDNA+ITS2 (flanked by 5.8S and 28SrDNA) were performed with the same partition, dividing as in the IQ-Tree analysis, with the following numbers of substitution types for each partition: nst2 for partitions 1 and 3, nst6 for partition 2. For all partitions, analysis was performed with Rates = InvGamma. Bayesian analysis was conducted with four Monte Carlo Markov chains running for 1 million generations, sampling every 100 generations (the first 25% of trees were discarded as ‘burn-in’).

The robustness of the ML trees generated by MEGA7 were assessed by bootstrap analysis with 1000 replicates (Felsenstein 1985). ML trees obtained with IQ-Tree were constructed under SH-aLRT (Guindon et al. 2010) and 1000 ultrafast bootstrap replicates (Minh et al. 2013; Hoang et al. 2018). Finally, BI trees were supported by posterior probability (PP) values. Bootstrap support values from ML analysis as well as PP values obtained on a 50% majority-rule consensus Bayesian tree were mapped onto the ML tree obtained by IQ-Tree. All the resulting trees were rooted with *Trochulus hispidus* sequences obtained from GenBank.

## ﻿Results

### ﻿Morphological study

Shell

**Figure 3.**
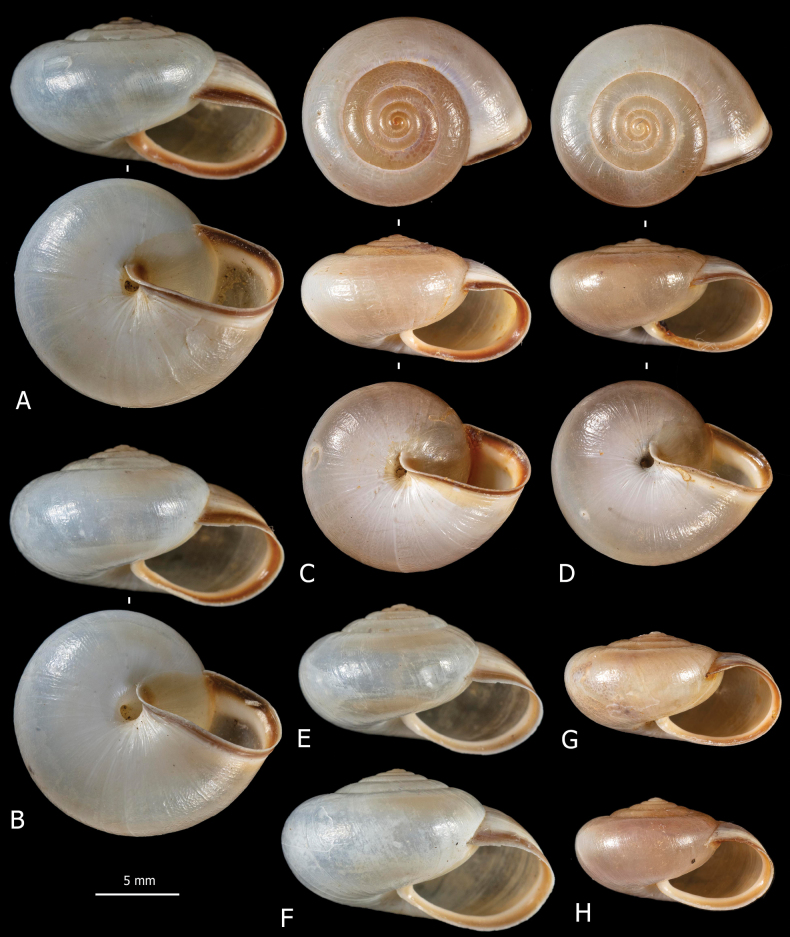
Shells of *Monacha cartusiana*. Specimens from Stazione di Castelnuovo Berardenga, G. Manganelli leg. 01.11.1981 (FGC 3430) (A, B, E, F) and Quattrovie, G. Manganelli leg. 24.09.2023 (FGC 55672) (C, D, G, H).

*Monacha claustralis* (Fig. 2A–H) and *Monacha cartusiana* (Fig. 3A–H) have rather fragile, subdiscoid to subglobose, sub-transparent shell, milky to yellowish in colour, with white and reddish collabral band near aperture; aperture rather large, oval to elliptical; umbilicus open, very small to small. Qualitative morphology reveals no evident differences in shell features between the two species.

**Figure 4.**
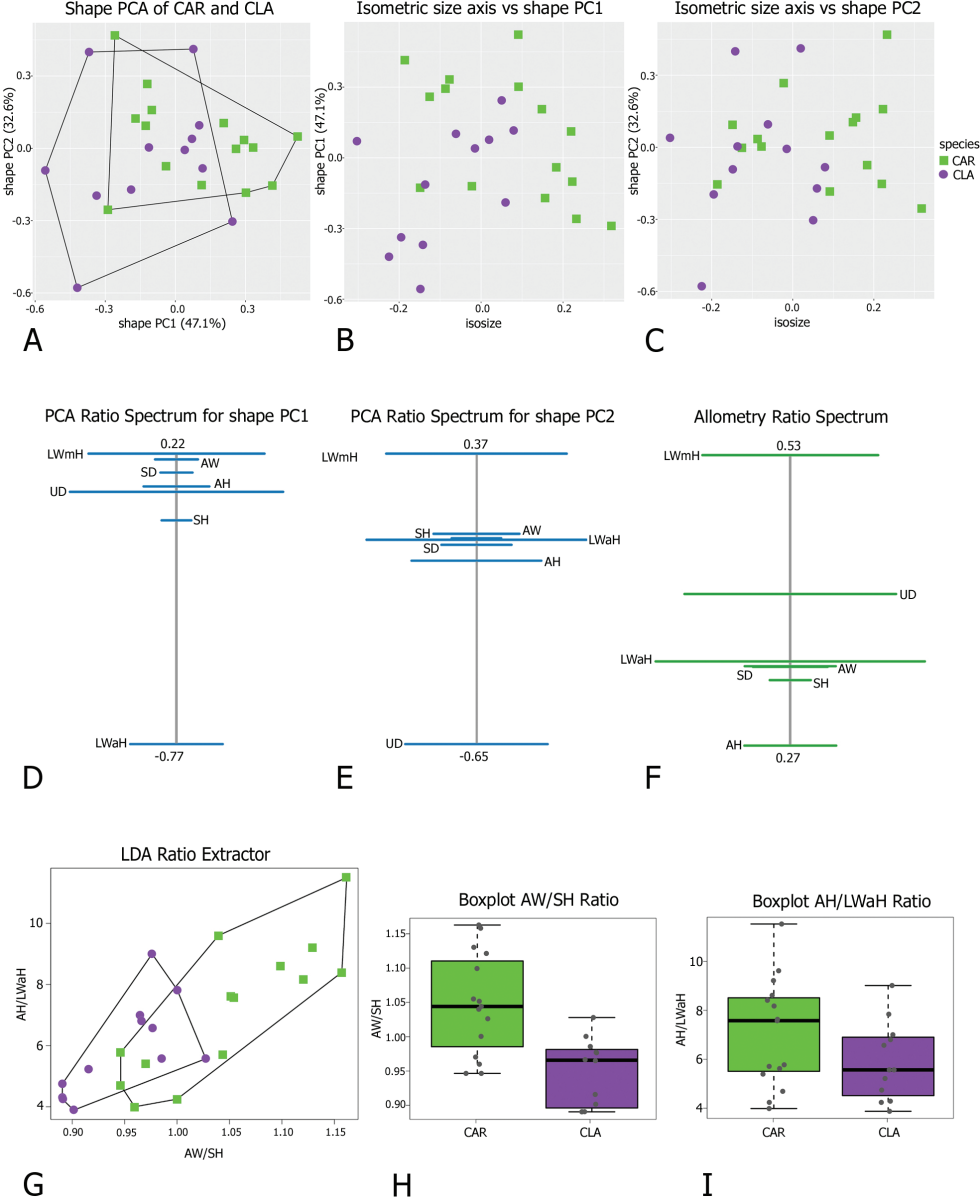
Morphometric analysis of *Monacha claustralis* (CLA) and *M. cartusiana* (CAR) shells. Scatterplot of principal component analysis (PCA) in shape space for shell variation in *Monacha cartusiana* (CAR) and *M. claustralis* (CLA) (A). Scatterplot of isometric size vs first and second principal components in shape space (B, C). PCA Ratio Spectrum of the first principal component. The ratio formed by the external points explains a large part of the variation of the first component. In contrast, ratios formed by characters lying close to each other in the spectrum explain very little (D). PCA Ratio Spectrum of the second principal component (E). Allometry Ratio Spectrum: horizontal bars in the ratio represent 68% bootstrap confidence intervals based on 999 replicates (F). Scatterplots of the two most discriminating ratios (AW/SH; AH/LWaH) for shells of CLA and CAR (G). Boxplots of AW/SH and AH/LWaH ratios (H, I).

Morphometric analysis of shell variation between the two species (Fig. 4A–I) suggested that neither size nor ratios are sufficient to clearly separate *M. claustralis* (CLA) and *M. cartusiana* (CAR). Shape PC1 explained 47.1% of the variance and was dominated by AW/LWaH and LWmH/LWaH ratios, as indicated by the position of these variables at opposite ends of the PCA Ratio Spectrum (Fig. 4D). Shape PC2 explained 32.6% of the variance and was mainly correlated with LWmH/UD ratio (Fig. 4E). The Allometry Ratio Spectrum (Fig. 4F) highlighted LWmH and AH as showing the greatest amount of allometry. The LDA Ratio Extractor (Fig. 4G) identified AW/SH as the most discriminating ratio for CAR and CLA. However, the overlapping ranges of AW/SH ratios (0.95–1.16 for CAR and 0.89–1.03 for CLA; Table 3) made them unsuitable for use in the identification key or diagnosis. The next best discriminating body ratio, i.e. the one least correlated with AW/SH, was AH/LWaH. Its standard distance was quite low (Dij = 1.56), compared to the higher standard distance Dij = 2.22 for AW/SH (Table 3). Again, the range of AH/LWaH overlapped for both species. Scatterplots (Fig. 4G) and boxplots (Fig. 4H, I) of the two most discriminating ratios confirmed that they lacked the power to separate the two species. Thus the analysis did not allow any further subdivision of CAR and CLA specimens on the basis of shell variability, at least with this dataset.

**Table 3.** First- and second-best ratios found by the LDA ratio extractor for separating shell data of *Monacha cartusiana* (CAR) and *M. claustralis* (CLA).

Group comparison	Best ratios	Range group 1	Range group 2	Standard distance	Delta value
CAR-CLA	AW/SH	0.95–1.16	0.89–1.03	2.22	0.32
CAR-CLA	AH/LWaH	4.00–11.5	4.25–9.00	1.56	0.40

**Figure 5.**
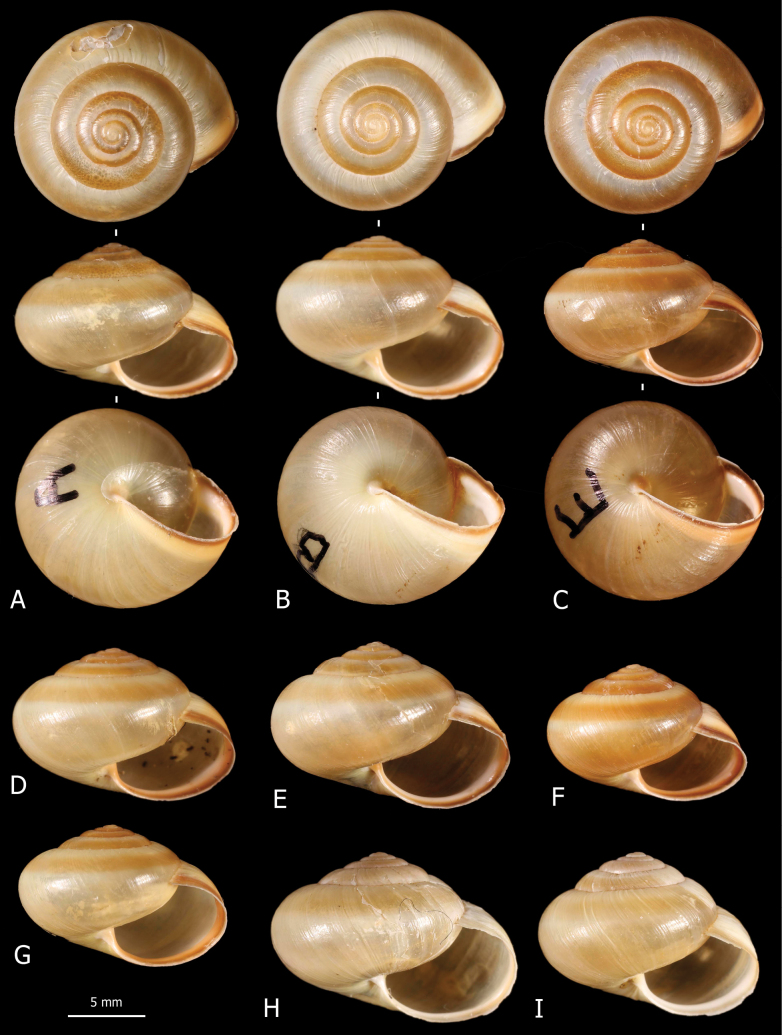
Shells of *Monacha parumcincta* from Corfu. Specimens from Paleokastritsa, along road to monastery of Paleokastritsa, A. Benocci, G. Manganelli and L. Manganelli leg. 25.10.2022 (FGC 52237) (A, D, G), Paleokastritsa, Bimbos supermarket, A. Benocci, G. Manganelli and L. Manganelli leg. 25.10.2022 (FGC 52237) (B, C, E, F) and Pantokrator, A. Benocci, G. Manganelli and L. Manganelli leg. 24.10.2022 (FGC 52237) (H, I).

**Figure 6.**
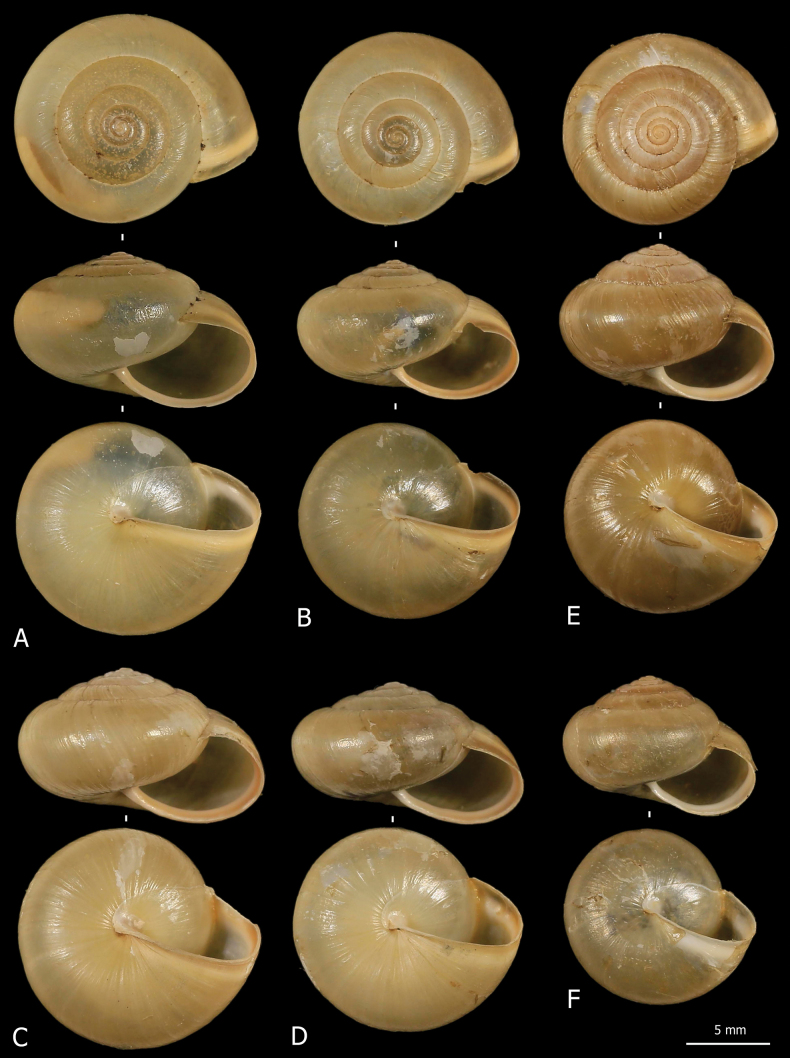
Shells of *Monacha parumcincta* from Italy. Specimens from A1 highway: rest area Romita est, S. Hallgass leg. 10.2013 (FGC 41561) (A, B), La Casella, G. Manganelli leg. 04.10.2015 (FGC 44077) (C, D), road Moliterno to Fontana d’Eboli, S. Hallgass leg. 10.2012 (FGC 42962) (E, F).

The shells of *Monacha parumcincta* populations from Corfu (Fig. 5A–I) and Italy (Fig. 6A–F) have rather robust, opaque, subglobose to globose shell, yellowish or brownish in colour with white and reddish collabral band near aperture; aperture rather large, round to oval; umbilicus closed by reflected columellar peristome. The most obvious differences between the two *parumcincta* groups are a glossier shell and variably evident whitish peripheral and subsutural bands in populations from Corfu and less glossy, more opaque shell and usually absent peripheral and subsutural bands in Italian populations.

**Figure 7.**
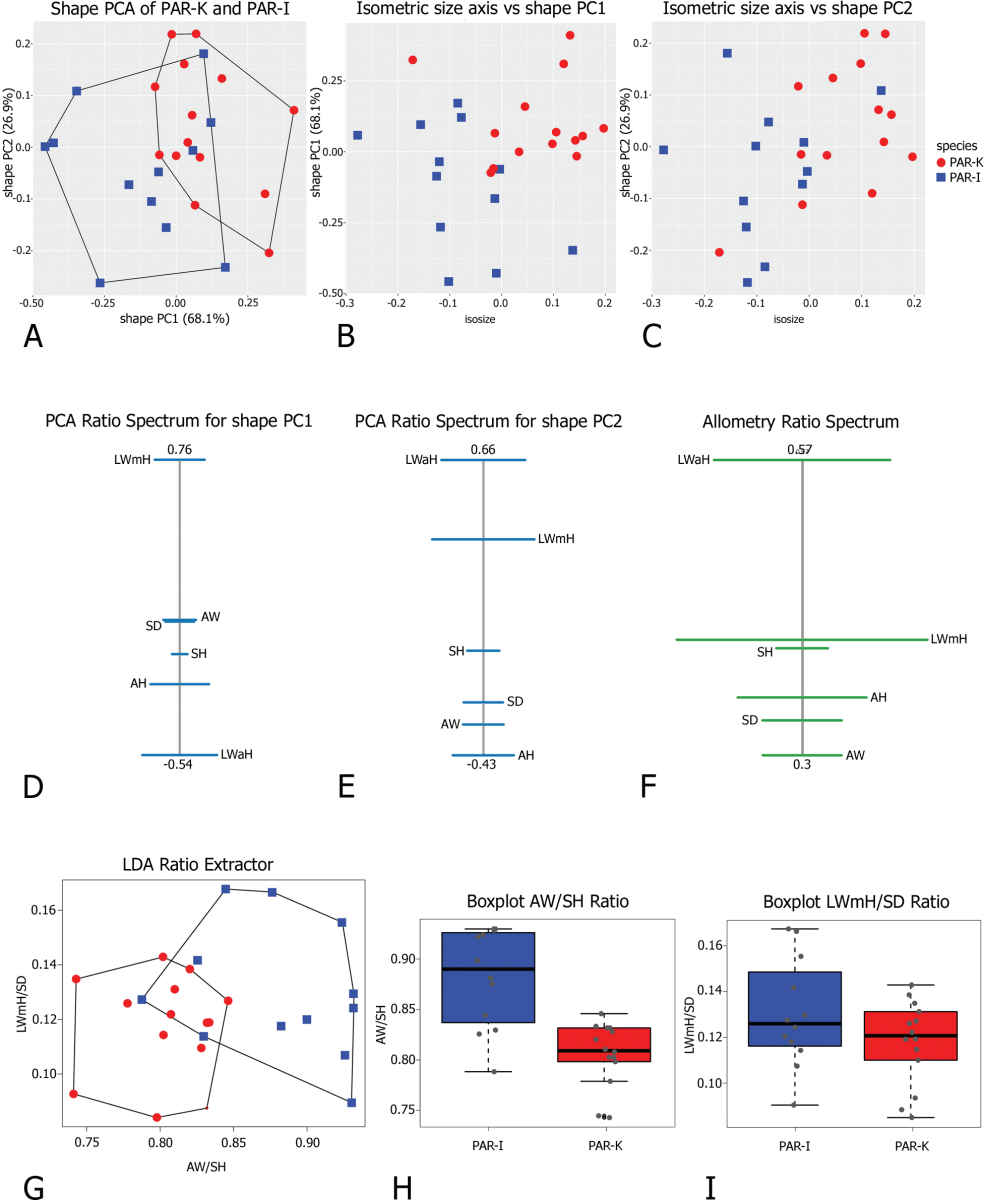
Morphometric analysis of Corfu (PAR-K) and Italian (PAR-I) *M. parumcincta* shells. Scatterplot of principal component analysis (PCA) in shape space for shell variation of *Monacha parumcincta* from Italy (PAR-I) and Corfu (PAR-K) (A). Scatterplot of isometric size vs first and second principal component in shape space (B, C). PCA Ratio Spectrum of the first principal component. The ratio formed by the external points explains a large part of the variation of the first component. In contrast, ratios formed by characters lying close to each other in the spectrum explain very little (D). PCA Ratio Spectrum of the second principal component (E). Allometry Ratio Spectrum: horizontal bars in the ratio represent 68% bootstrap confidence intervals based on 999 replicates (F). Scatterplots of the two most discriminating ratios (AW/SH; LWmH/SD) for shell variation of PAR-I and PAR-K (G). Boxplots of AW/SH and LWmH/SD ratios (H, I).

Morphometric analysis of shell variation between Corfu (PAR-K) and Italian (PAR-I) *M. parumcincta* populations revealed no clear separation based on the plots of the two-first shape PCs (Fig. 7A) or the isometric size against shape PC1 (Fig. 7B): shape PC1 explained 68.1% of the variance and was dominated by the LWmH/LWaH ratio as shown by the PCA Ratio Spectrum of shape PC1 (Fig. 7D). Shape PC2 explained 26.9% of the variance and was mainly correlated with ratios like LWaH/AH (Fig. 7E). The Allometry Ratio Spectrum indicated the greatest amount of allometry for LWaH and AW (Fig. 7F). The LDA Ratio Extractor (Fig. 7G) identified AW/SH as the most discriminating ratio but overlapping ranges (0.78–0.93 for PAR-I; 0.74–0.83 for PAR-K; Table 4) excluded it for use in the identification key and diagnosis. Similarly, LWmH/SD, with a standard distance of 2.09 compared with the standard distance Dij = 2.81 (Table 4) of the first ratio, failed to provide clear separation due to overlapping ranges. Scatterplot (Fig. 7G) and boxplots (Fig. 7H, I) confirmed the limited discriminating power of both ratios, demonstrating that the dataset did not admit further subdivision of shell variability in the study populations.

**Table 4.** First- and second-best ratios found by the LDA ratio extractor for separating shell data of *Monacha parumcincta* from Corfu (PAR-K) and Italy (PAR-I).

Group comparison	Best ratios	Range group 1	Range group 2	Standard distance	Delta value
PAR-I – PAR-K	AW/SH	0.78–0.93	0.74–0.84	2.81	0.35
PAR-I – PAR-K	LWmH/SD	0.08–0.17	0.08–0.14	2.09	0.42

#### ﻿Anatomy

**Figure 8.**
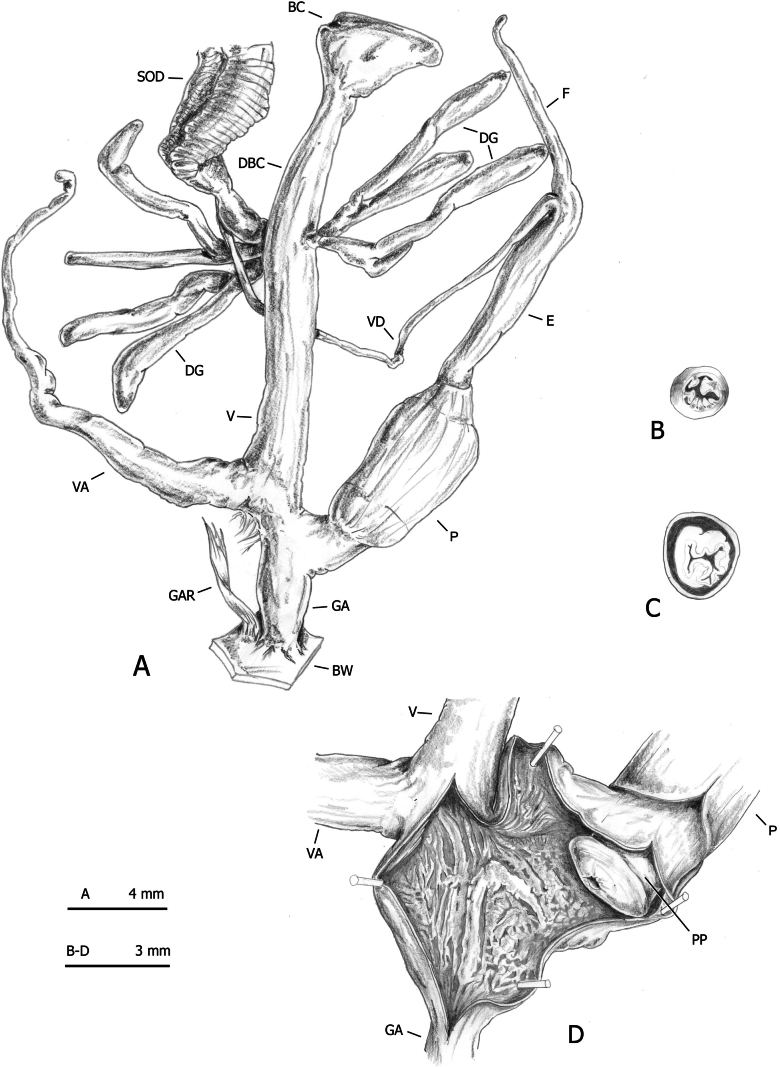
Distal genitalia of *Monacha claustralis* from Corfu (Kérkyra). Specimen from Molos cemetery, A. Benocci, G. Manganelli and L. Manganelli leg. 26.10.2022 (FGC 52301). Distal genitalia (A), transverse sections of medial epiphallus (B) and apical penial papilla (C), internal structure of distal genitalia (D).

**Figure 9.**
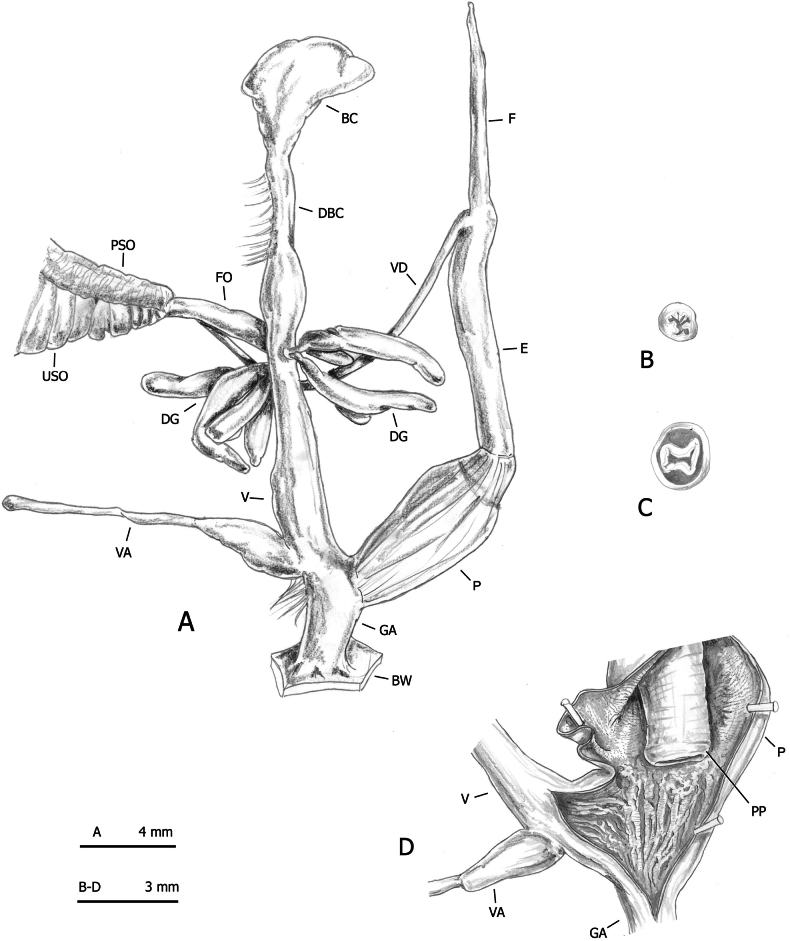
Distal genitalia of *Monacha claustralis* from Corfu (Kérkyra). Specimen from Gardiki, A. Benocci, G. Manganelli and L. Manganelli leg. 26.10.2022 (FGC 52297). Distal genitalia (A), transverse sections of medial epiphallus (B) and apical penial papilla (C), internal structure of distal genitalia (D).

**Figure 10.**
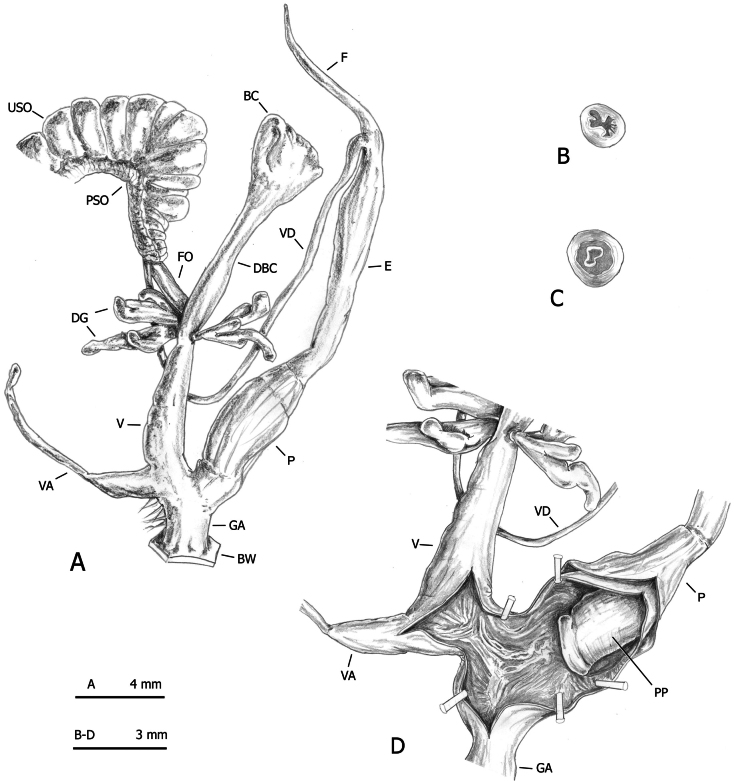
Distal genitalia of *Monacha claustralis* from Corfu (Kérkyra). Specimen from Benitses, A. Benocci, G. Manganelli and L. Manganelli leg. 24.10.2022 (FGC 52237). Distal genitalia (A), transverse sections of medial epiphallus (B) and apical penial papilla (C), internal structure of distal genitalia (D).

**Figure 11.**
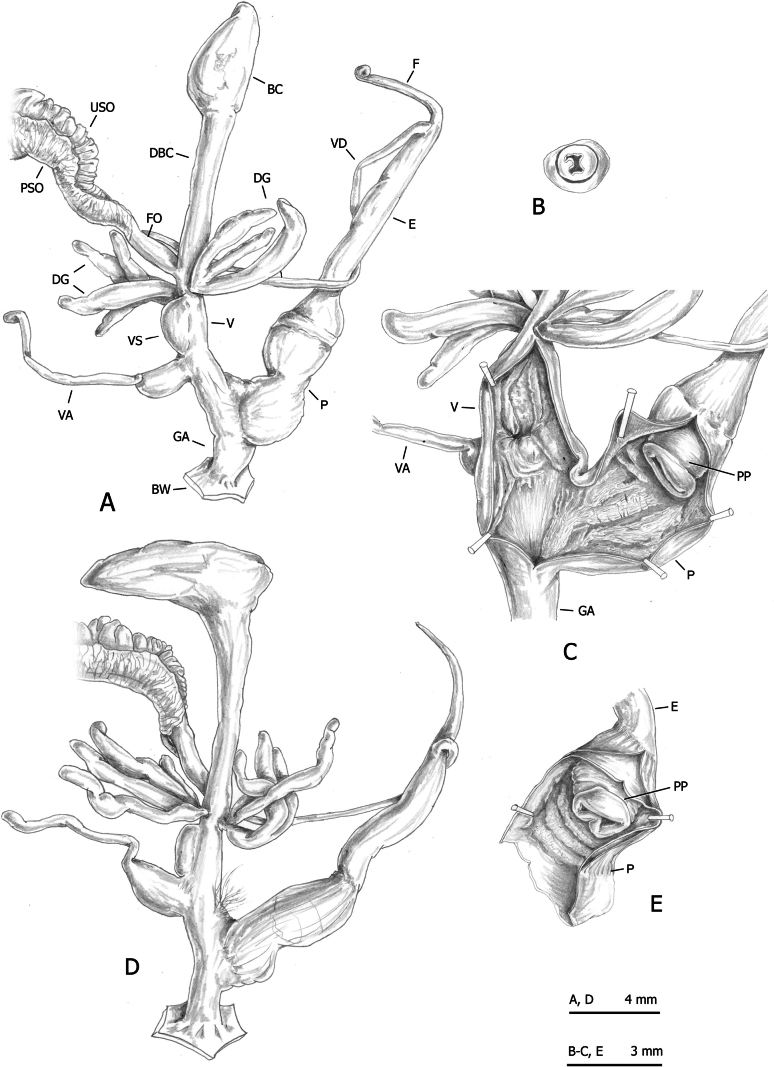
Genital anatomy of *Monacha cartusiana*. Specimens from Quattrovie, G. Manganelli leg. 24.09.2023 (FGC 55672) (A–C) and Stazione di Castelnuovo Berardenga, G. Manganelli leg. 01.11.1981 (FGC 3430) (D, E). Distal genitalia (A, D), transverse section of apical penial papilla (B), internal structure of distal genitalia (C) and of penis (E).

Both groups *Monacha claustralis* (Figs 8–10) and *Monacha cartusiana* (Fig. 11) on one hand, and *Monacha parumcincta* from Corfu (Figs 13–15) and from Italy (Fig. 16) on the other, had very similar structure of the distal genitalia. They all featured digitiform glands and vaginal appendix while lacking penial retractor, characters which are typical of species of the nominotypical subgenus according to the taxonomy proposed by Neiber and Hausdorf (2017).

The main differences between the first two species (*Monacha claustralis* and *Monacha cartusiana*) concerned the distal vagina, lateral vaginal sac, and vaginal appendix (distal vagina long; lateral vaginal sac absent; vaginal appendix inserted near distal end of vagina in *M. claustralis* vs distal vagina short; lateral vaginal sac present; vaginal appendix inserted approximately half-way along vagina in *M. cartusiana*).

**Figure 12.**
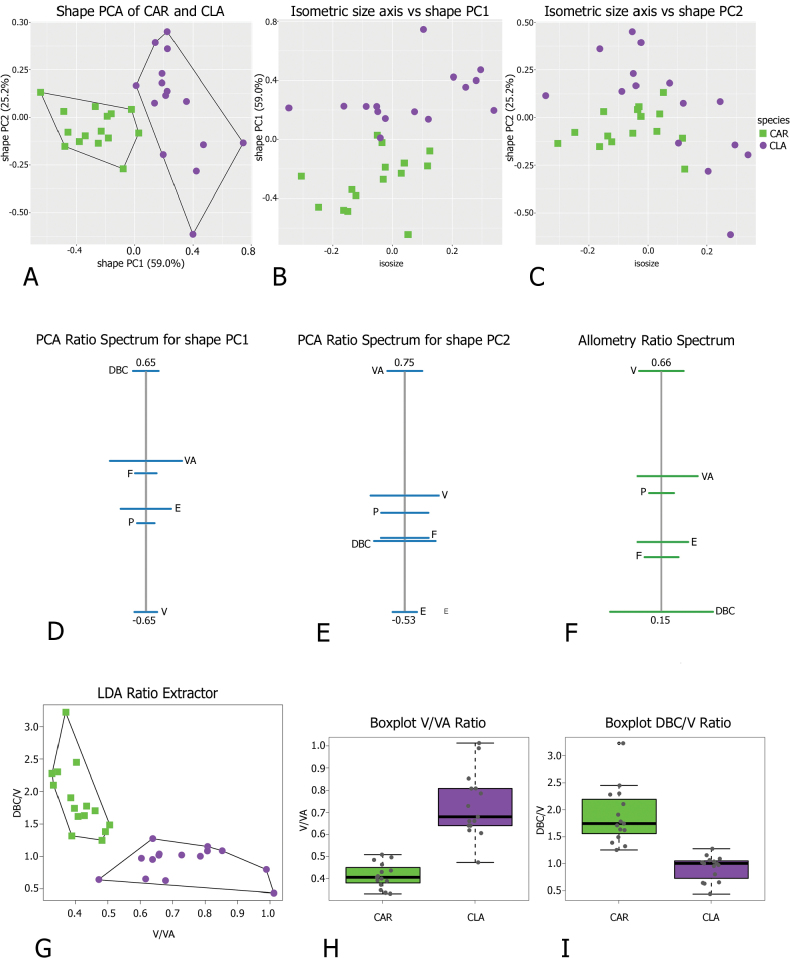
Morphometric analysis of *Monacha claustralis* (CLA) and *M. cartusiana* (CAR) distal genitalia. Scatterplot of principal component analysis (PCA) in shape space for genital variations in *Monacha cartusiana* (CAR) and *M. claustralis* (CLA) (A). Scatterplot of isometric size vs first and second principal component in shape space (B, C). PCA Ratio Spectrum of the first principal component. The ratio formed by the external points explains a large part of the variation of the first component. In contrast, ratios formed by characters lying close to each other in the spectrum explain very little (D). PCA Ratio Spectrum of the second principal component (E). Allometry Ratio Spectrum: horizontal bars in the ratio represent 68% bootstrap confidence intervals based on 999 replicates (F). Scatterplots of the two most discriminating ratios (V/VA; DBC/V) for genitalia of CAR and CLA (G). Boxplots of V/VA and DBC/V ratios (H, I).

Concerning genital variation, a scatterplot of the first two shape PCs (Fig. 12A) showed distinct positions of *M. claustralis* (CLA) and *M. cartusiana* (CAR) along PC1, as the two clusters did not overlap. Shape PC1 explained 59% of the variance and was dominated by the DBC/V ratio, as indicated by the position of these variables at opposite ends of the PCA Ratio Spectrum for shape PC1 (Fig. 12D). In contrast, shape PC2 was dominated by the VA/E ratio (Fig. 12E). Scatterplots revealed a possible correlation between isosize and shape PC1 (Fig. 12B; Pearson’s product-moment correlation: t = 2.9668, df = 28, p-value = 0.01, cor = 0.49), but a weaker correlation for shape PC2 (Fig. 12C; t = -2.1157, df = 28, p-value = 0.04, cor = -0.37). Analysis of the Allometry Ratio Spectrum sustained the idea that some shape components are influenced by dimensional variations, suggesting a potential allometric effect (Fig. 12F). The DBC/V ratio, dominant in the PCA Ratio Spectrum for PC1, provided indirect evidence of allometric behaviour, as it aligned with the V/DBC ratio identified in the Allometric Ratio Spectrum as the most significant for capturing size-related variation. The LDA Ratio Extractor (Fig. 12G) indicated V/VA as the ratio that best discriminates CLA and CAR in terms of genital variation. The delta measurement (δ), which indicates how well shape discriminates in relation to size (δ close to 1 suggests that separation is mainly size-driven, whereas δ close to 0 suggests shape-based separation), was 0.15 for the V/VA ratio (Table 5). This indicated that the separation of groups was primarily driven by shape differences rather than size (as further demonstrated by D_size_ = 0.184 and D_shape_ = 0.997). Most CAR specimens had a V/VA ratio < 0.5 (0.33–0.50), whereas most CLA individuals had a V/VA ratio > 0.6, with more variability in this range (0.47–1.01) (Fig. 12G, H, I; Table 5). The next best discriminating body ratio, selected for being as little correlated as possible with V/VA, was DBC/V. Its standard distance (Dij) was 2.32, which is lower than the relatively high Dij = 4.07 of the V/VA ratio (Table 5). The delta (δ) value for DBC/V was 0.24, slightly higher than for V/VA but still close to zero, again indicating a predominant effect of shape. DBC/V may be influenced by size, but its low δ value indicated that shape remained the predominant factor in discrimination. However, the standard distance of 2.32 suggested that while useful for supporting separation, DBC/V alone may not provide very reliable identification. It was more appropriate as a complementary ratio, supporting the primary discriminating ratio V/VA, which showed a stronger capacity to distinguish the groups.

**Table 5.** First- and second-best ratios found by the LDA ratio extractor for separating genital data of *Monacha cartusiana* (CAR) and *M. claustralis* (CLA).

Group comparison	Best ratios	Range group 1	Range group 2	Standard distance	Delta value
CAR-CLA	V/VA*	0.33–0.50	0.47–1.01	4.07	0.15
CAR-CLA	DBC/V*	1.25–3.22	0.43–1.15	2.32	0.24

Ratios marked with * have very little or no overlap and therefore are suitable in the identification key and diagnoses.

**Figure 13.**
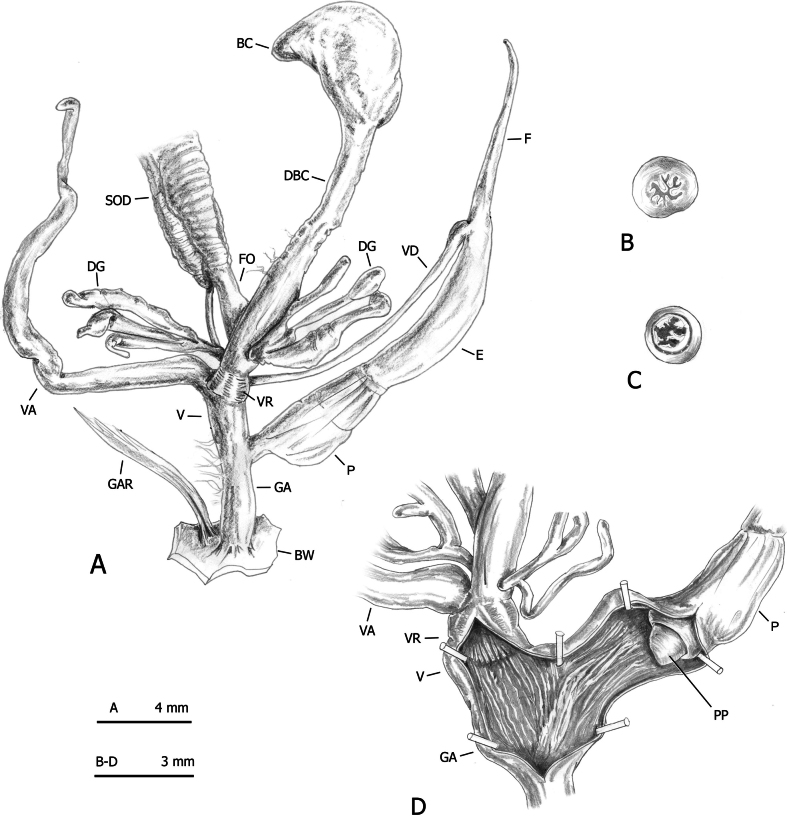
Distal genitalia of *Monacha parumcincta* from Corfu (Kérkyra). Specimen from Paleokastritsa, along road to monastery of Paleokastritsa, A. Benocci, G. Manganelli and L. Manganelli leg. 25.10.2022 (FGC 52237). Distal genitalia (A), transverse sections of medial epiphallus (B) and apical penial papilla (C), internal structure of distal genitalia (D).

**Figure 14.**
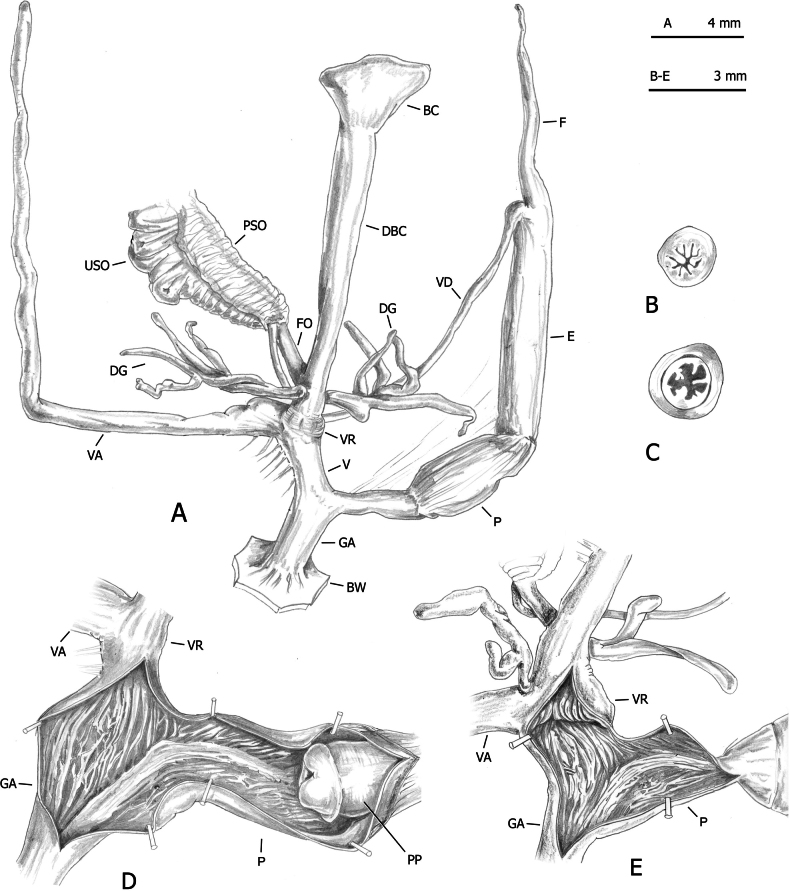
Distal genitalia of *Monacha parumcincta* from Corfu (Kérkyra). Specimens from Paleokastritsa, Bimbos supermarket, A. Benocci, G. Manganelli and L. Manganelli leg. 25.10.2022 (FGC 52237). Distal genitalia (A), transverse sections of medial epiphallus (B) and apical penial papilla (C), internal structure of distal genitalia (D, E).

**Figure 15.**
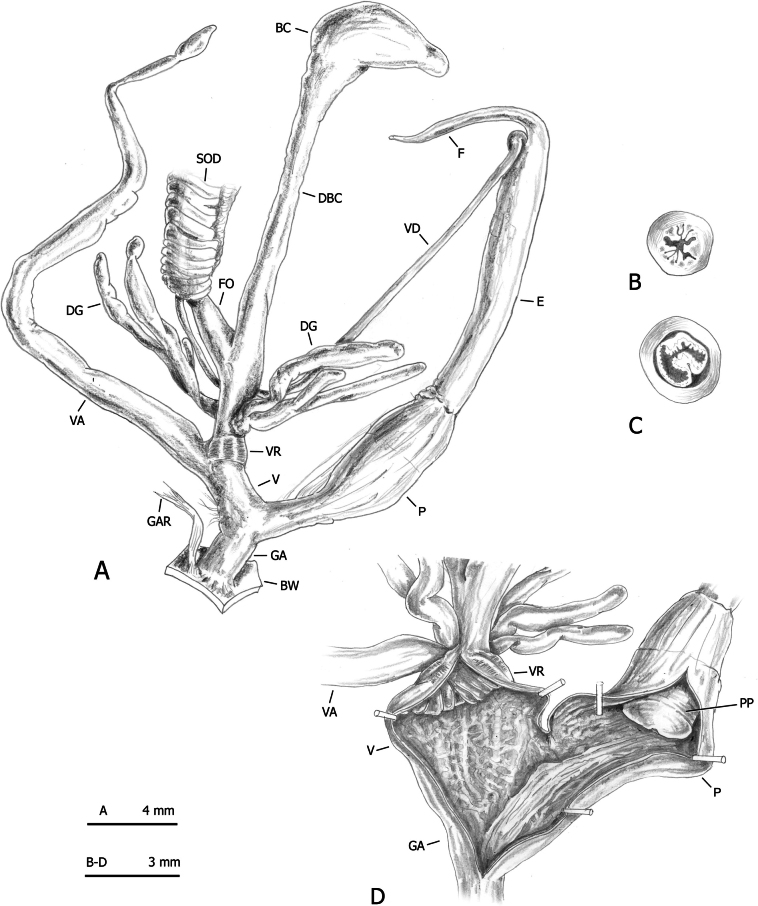
Distal genitalia of *Monacha parumcincta* from Corfu (Kérkyra). Specimen from Pantokrator, A. Benocci, G. Manganelli and L. Manganelli leg. 24.10.2022 (FGC 52237). Distal genitalia (A), transverse sections of medial epiphallus (B) and apical penial papilla (C), internal structure of distal genitalia (D).

**Figure 16.**
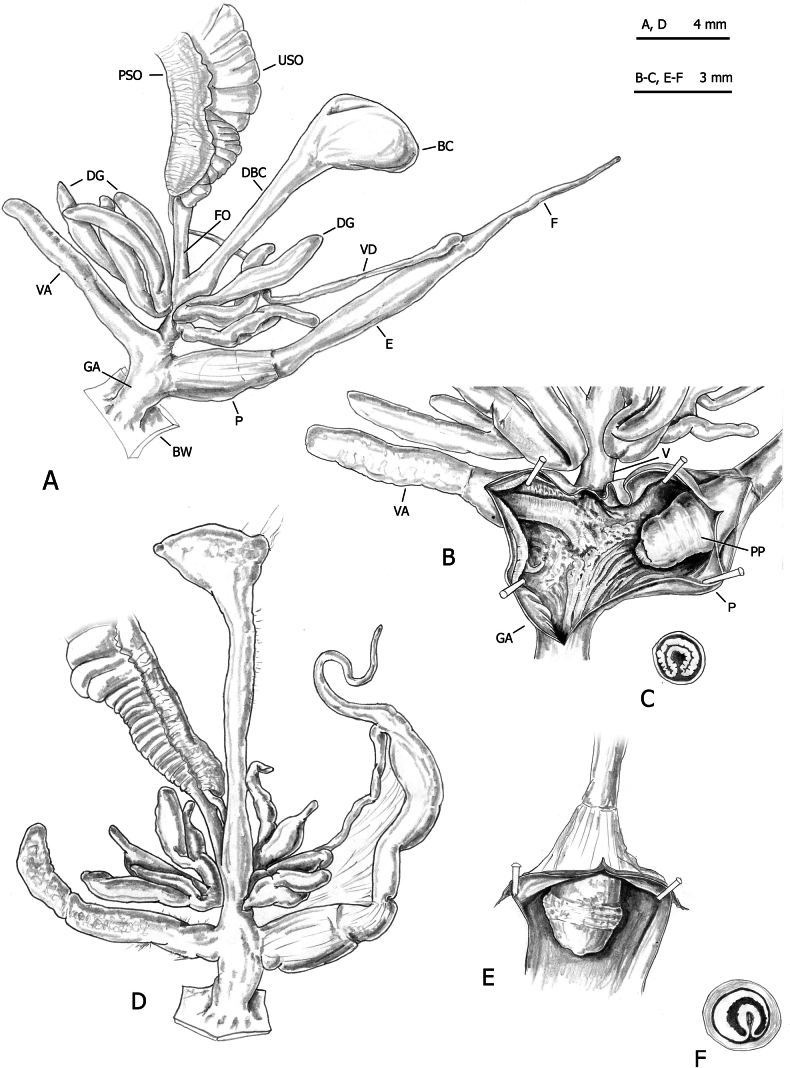
Genital anatomy of *Monacha parumcincta* from Italy. Specimens from road Moliterno to Fontana d’Eboli, S. Hallgass leg. 10.2012 (FGC 42962) (A–C) and La Casella, G. Manganelli leg. 04.10.2015 (FGC 44077) (D–F). Distal genitalia (A, D), internal structure of distal genitalia (B) and of penis (E), transverse sections of apical penial papilla (C, F).

The main differences between the populations of *Monacha parumcincta* from Corfu (Figs 13–15) and Italy (Fig. 16) concerned vaginal length, refringent ring, vaginal appendix and transverse section of central duct of penial papilla (vagina very short to short; refringent ring present; vaginal appendix very long, inserted approximately half-way along vagina, without dilated basal portion and progressively tapering towards tip; transverse section of central duct of penial papilla round in Corfu populations vs vagina rather long to long; refringent ring absent; vaginal appendix rather short, inserted near distal end of vagina without dilated basal portion and of uniformly wide calibre; transverse section of central duct of penial papilla C-shaped in Italian populations).

**Figure 17.**
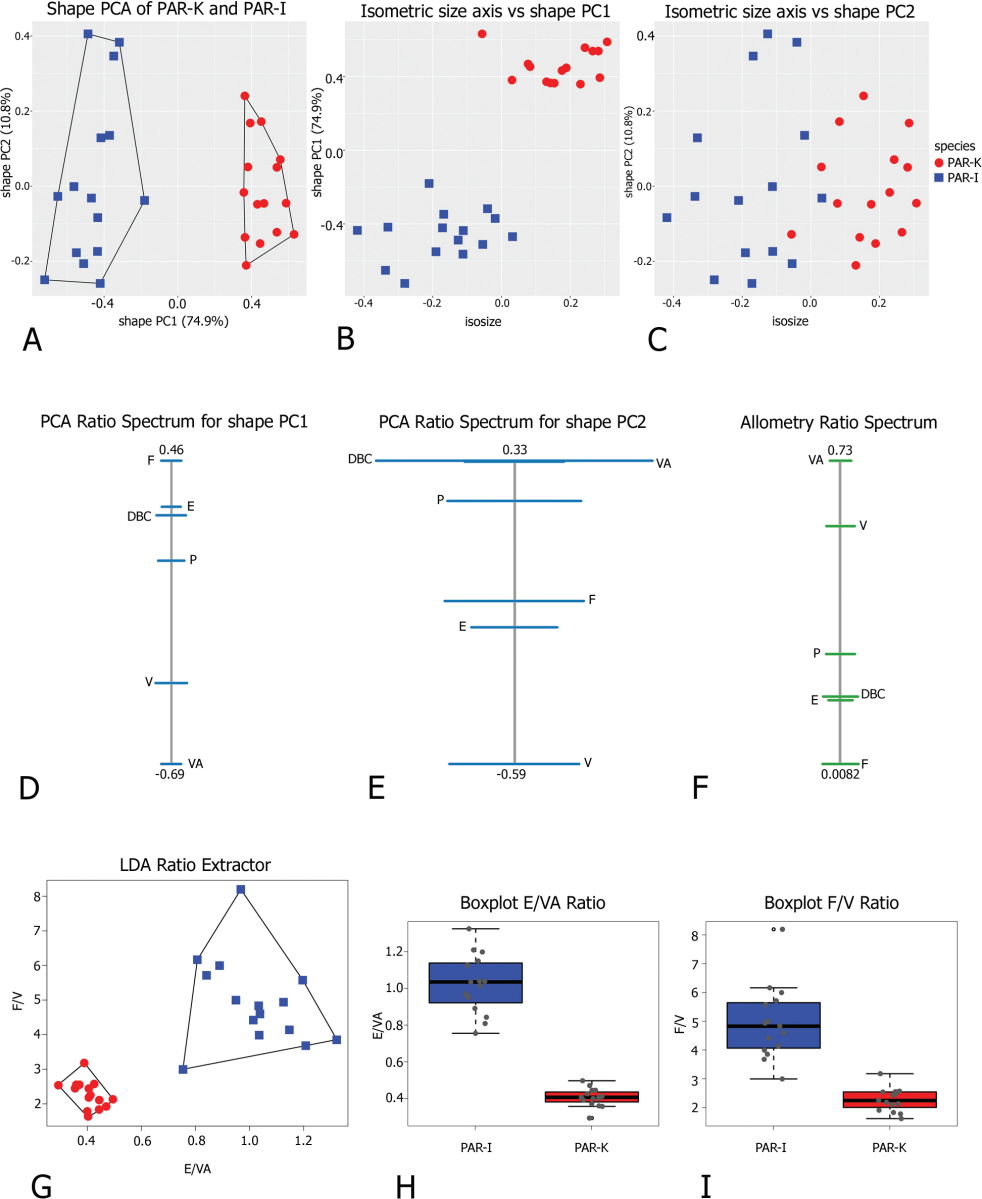
Morphometric analysis of distal genitalia of Corfu (PAR-K) and Italian (PAR-I) *Monacha parumcincta*. Scatterplot of principal component analysis (PCA) in shape space for genital variations in *Monacha parumcincta* from Italy (PAR-I) and Corfu (PAR-K) (A). Scatterplot of isometric size vs first and second principal components in shape space (B, C). PCA Ratio Spectrum of the first principal component. The ratio formed by the external points explains a large part of the variation of the first component. In contrast, ratios formed by characters lying close to each other in the spectrum explain very little (D). PCA Ratio Spectrum of the second principal component (E). Allometry Ratio Spectrum: horizontal bars in the ratio represent 68% bootstrap confidence intervals based on 999 replicates (F). Scatterplots of the two most discriminating ratios (E/VA; F/V) for genitalia of PAR-I and PAR-K (G). Boxplots of E/VA and F/V ratios (H, I).

Morphometric analysis of genital variation (Fig. 17A–I) between Corfu (PAR-K) and Italian (PAR-I) *M. parumcincta* populations revealed that shape PC1 was fully congruent with the separation of PAR-I and PAR-K (Fig. 17A): PC1 alone explained 74.9% of the variance and was dominated by ratios like F/VA, as indicated by the position of these variables at opposite ends of the PCA Ratio Spectrum (Fig. 17D). To assess the amount of allometry, the isometric size axis was plotted against shape PC1 to see how strongly shape correlated with size (Fig. 17B); a similar trend emerged when we compared the PCA Ratio Spectrum (Fig. 17D) and the Allometry Ratio Spectrum (Fig. 17F): the ratio of the major variables for shape PC1 (VA and F) was also the most allometric, confirming a certain amount of size-related variation. The LDA Ratio Extractor (Fig. 17G) showed that E/VA was the ratio that best discriminated PAR-I and PAR-K. The delta measurement was 0.243, indicating that discrimination between groups stems mostly from shape differences (as also demonstrated by D_size_ = 0.295; D_shape_ = 0.92 values). Most PAR-K have an E/VA ratio < 0.5 (0.35–0.49) whereas most PAR-I have an E/VA ratio > 0.8, with more variability within this range (0.75–1.32) (Table 6). The next discriminating body ratio, as little correlated as possible with E/VA, was F/V. Its standard distance Dij was 6.43 compared to the relatively high standard distance Dij = 9.15 of the first ratio (Table 6). As shown also by the scatterplot (Fig. 17G), the discriminating power was lower than that of the first ratio (with very little overlap of ranges, see Table 6).

**Table 6.** First- and second-best ratios found by the LDA ratio extractor for separating genital data of *Monacha parumcincta* from Corfu (PAR-K) and Italy (PAR-I).

Group comparison	Best ratios	Range group 1	Range group 2	Standard distance	Delta value
PAR-I – PAR-K	E/VA*	0.75–1.32	0.35–0.49	9.15	0.24
PAR-I – PAR-K	F/V*	3.00–8.20	1.60–3.20	6.43	0.31

Ratios marked with * have very little or no overlap and therefore are suitable in the identification key and diagnoses.

### ﻿Molecular study

One hundred seventy two new sequences obtained by molecular analysis were deposited in GenBank: 51 of COI (PP947873–PP947923), 60 of 16SrDNA (PP949387–PP949446), and 61 of ITS2 with flanking fragments of 5.8SrDNA and 28SrDNA (PP947951–PP948011 (Table 1)). Among them, 25 haplotypes of COI, 38 haplotypes of 16SrDNA, and 17 haplotypes of ITS2 with flanks were identified (Table 1). These haplotypes were used for phylogenetic analysis based on single gene sequences and concatenated mitochondrial and nuclear gene data sets of sequences.

**Figure 18.**
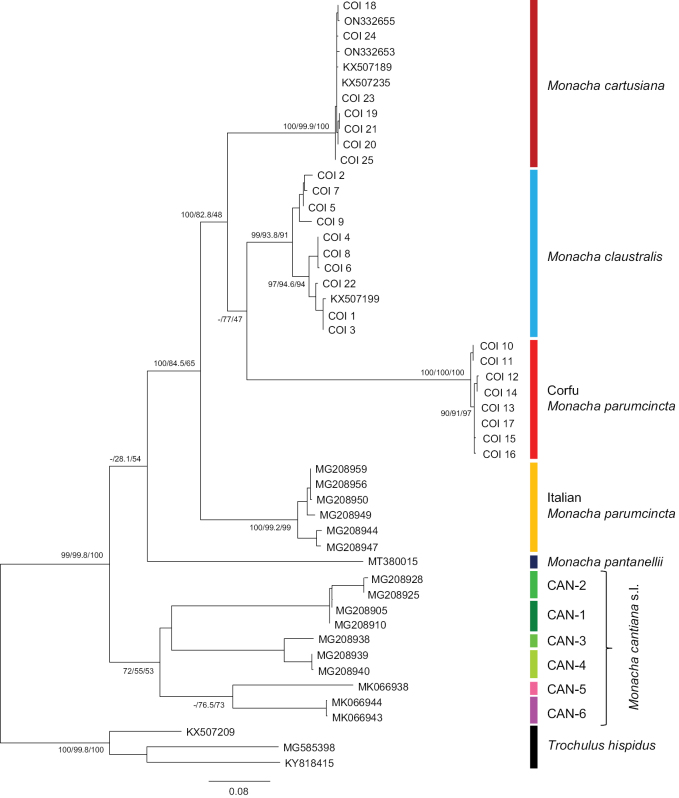
Maximum Likelihood (ML) tree of COI haplotypes of *Monacha claustralis* and *M. parumcincta* from Corfu (Kérkyra). COI sequences of *M. claustralis* and *M. parumcincta* from Corfu (Table 1) were compared with new COI sequences of *M. cartusiana* as well as with COI sequences of Italian *M. parumcincta*, *M. pantanellii* and *M. cantiana* s.l. obtained from GenBank (Table 1). Numbers next to main branches indicate (left to right): bootstrap supports above 50% calculated by ML-MEGA7 (Kumar et al. 2016) on 1000 replicates (Felsenstein 1985), SH-aLRT and ultrafast bootstrap in IQ-Tree (Nguyen et al. 2015). The tree was rooted with *Trochulus hispidus* sequences deposited in GenBank by Neiber and Hausdorf 2015, 2017 (KX507209), Neiber et al. 2017 (KY818415) and Caro et al. 2019 (MG585398).

The phylogenetic analysis of COI sequences obtained from the specimens studied and comparative sequences from GenBank is shown in Fig. 18. The COI sequences were grouped in four well-supported clades that can be assigned to *M. cartusiana* (haplotypes COI 18 – COI 21, COI 23 – COI 25 together with KX507189, KX507235, ON332653, ON332655 deposited for *M. cartusiana* in GenBank), *M. claustralis* (haplotypes COI 1 – COI 9, COI 22 with KX507199 from GenBank), Corfu *M. parumcincta* (haplotypes COI 10 – COI 17), and Italian *M. parumcincta* (GenBank sequences MG208944, MG208947, MG208949, MG208950, MG208956, MG208959). These four groups were also separated from sequences of *M. pantanellii* (MT380015) and six lineages of *M. cantiana* s.l. (CAN-1 MG208905, MG208910; CAN-2 MG208925, MG208928; CAN-3 MG208938; CAN-4 MG208939, MG208940; CAN-5 MK066938; CAN-6 MK066943, MK066944).

K2P genetic distances (Table 7) showed small genetic differentiation between COI sequences in four species, i.e. in *M. claustralis*, Corfu *M. parumcincta*, Italian *M. parumcincta*, and *M. cartusiana* (mean distance 0.4–3.3%). On the other hand, K2P distances between these four species as well as between them and *M. pantanellii* or six lineages of *M. cantiana* s.l. were much larger (from a mean of 14.0% between *M. claustralis* and *M. cartusiana* to 21.2% between *M. claustralis* and *M. cantiana* s.l. CAN-2). It is noteworthy that the genetic distances were usually more than 18%.

**Table 7.** Ranges of K2P genetic distances between the COI sequences analysed (mean value in brackets).

Comparison	COI (%)
Within *M. claustralis*	0.0–5.6 (3.3)
Within Corfu *M. parumcincta*	0.0–1.1 (0.5)
Within Italian *M. parumcincta*	0.0–4.5 (2.6)
Within *M. cartusiana*	0.0–0.7 (0.4)
Between *M. claustralis* and Corfu *M. parumcincta*	14.5–17.4 (16.1)
Between *M. claustralis* and Italian *M. parumcincta*	13.8–15.9 (15.0)
Between *M. claustralis* and *M. cartusiana*	13.2–15.3 (14.0)
Between *M. claustralis* and *M. cantiana* CAN-1	20.0–22.4 (21.0)
Between *M. claustralis* and *M. cantiana* s.l. CAN-2	20.4–22.4 (21.2)
Between *M. claustralis* and *M. cantiana* s.l. CAN-3	18.2–19.7 (18.9)
Between *M. claustralis* and *M. cantiana* s.l. CAN-4	16.3–17.4 (16.9)
Between *M. claustralis* and *M. cantiana* s.l. CAN-5	19.5–20.3 (20.0)
Between *M. claustralis* and *M. cantiana* s.l. CAN-6	16.5–18.3 (17.1)
Between *M. claustralis* and *M. pantanellii*	17.1–18.6 (17.9)
Between Corfu *M. parumcincta* and Italian *M. parumcincta*	18.7–20.1 (19.4)
Between Corfu *M. parumcincta* and *M. cartusiana*	17.0–18.9 (17.9)
Between Corfu *M. parumcincta* and *M. cantiana* CAN-1	19.7–21.0 (20.3)
Between Corfu *M. parumcincta* and *M. cantiana* s.l. CAN-2	20.4–21.5 (21.0)
Between Corfu *M. parumcincta* and *M. cantiana* s.l. CAN-3	19.0–19.5 (19.1)
Between Corfu *M. parumcincta* and *M. cantiana* s.l. CAN-4	19.0–19.4 (19.2)
Between Corfu *M. parumcincta* and *M. cantiana* s.l. CAN-5	19.7–20.1 (19.8)
Between Corfu *M. parumcincta* and *M. cantiana* s.l. CAN-6	16.1–16.7 (16.4)
Between Corfu *M. parumcincta* and *M. pantanellii*	16.8–17.9 (17.7)
Between Italian *M. parumcincta* and *M. cartusiana*	15.0–16.7 (15.7)
Between Italian *M. parumcincta* and *M. cantiana* CAN-1	20.1–21.1 (20.7)
Between Italian *M. parumcincta* and *M. cantiana* s.l. CAN-2	19.6–20.6 (20.1)
Between Italian *M. parumcincta* and *M. cantiana* s.l. CAN-3	18.7–20.4 (19.1)
Between Italian *M. parumcincta* and *M. cantiana* s.l. CAN-4	19.5–20.4 (20.0)
Between Italian *M. parumcincta* and *M. cantiana* s.l. CAN-5	18.2–20.1 (18.8)
Between Italian *M. parumcincta* and *M. cantiana* s.l. CAN-6	17.4–18.0 (17.7)
Between Italian *M. parumcincta* and *M. pantanellii*	19.2–19.8 (19.6)
Between *M. cartusiana* and *M. cantiana* CAN-1	18.9–19.9 (19.4)
Between *M. cartusiana* and *M. cantiana* s.l. CAN-2	20.6–21.3 (20.9)
Between *M. cartusiana* and *M. cantiana* s.l. CAN-3	19.3–20.2 (19.7)
Between *M. cartusiana* and *M. cantiana* s.l. CAN-4	18.4–18.9 (18.7)
Between *M. cartusiana* and *M. cantiana* s.l. CAN-5	20.8–21.2 (21.0)
Between *M. cartusiana* and *M. cantiana* s.l. CAN-6	17.1–17.6 (17.3)
Between *M. cartusiana* and *M. pantanellii*	18.1–18.7 (18.4)

**Figure 19.**
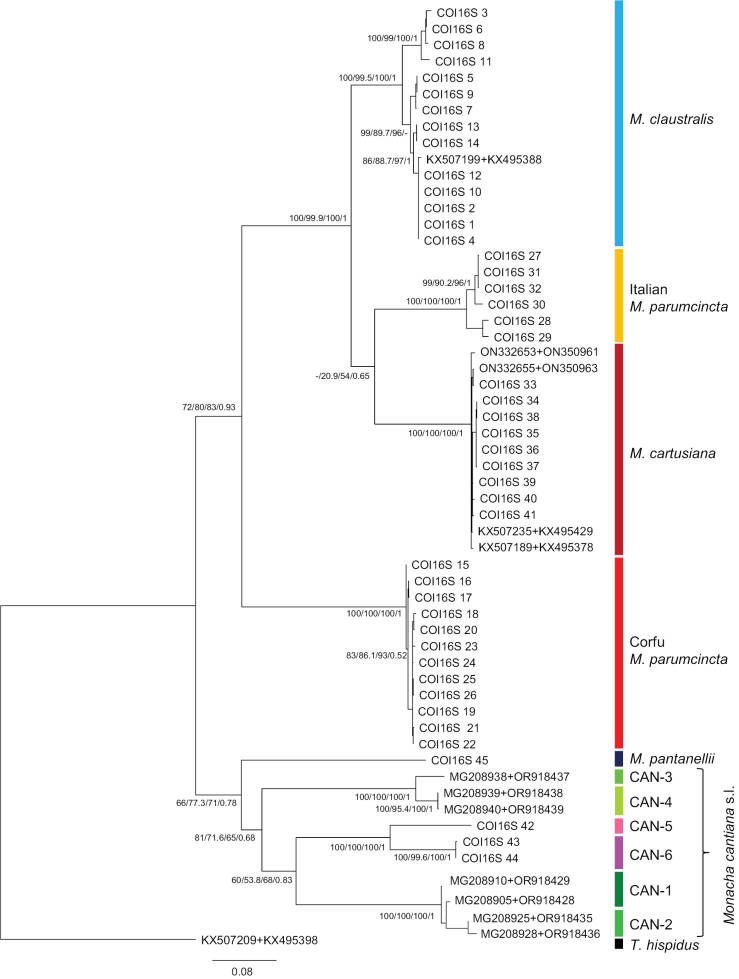
Maximum Likelihood (ML) tree of concatenated sequences of COI and 16SrDNA haplotypes of *Monacha claustralis* and *M. parumcincta* from Corfu (Kérkyra). COI and 16SrDNA sequences of *M. claustralis* and *M. parumcincta* from Corfu (Table 1) were compared with new COI and 16SrDNA sequences of *M. cartusiana* as well as with COI and 16SrDNA sequences of Italian *M. parumcincta*, *M. pantanellii* and *M. cantiana* s.l. obtained from GenBank (Tables 1, 2). Numbers next to main branches indicate (left to right): bootstrap supports above 50% calculated by ML-MEGA7 (Kumar et al. 2016) on 1000 replicates (Felsenstein 1985), SH-aLRT and ultrafast bootstrap in IQ-Tree (Nguyen et al. 2015), and posterior probabilities by BI (Ronquist et al. 2012). The tree was rooted with *Trochulus hispidus* concatenated sequences obtained from GenBank (Table 2).

Similar phylogenetic results were obtained in the case of 16SrDNA sequences (not shown) and concatenated sequences of mitochondrial genes COI+16SrDNA (Fig. 19, Table 2). These analyses showed separateness of four species (*M. claustralis*, Italian *M. parumcincta*, *M. cartusiana*, Corfu *M. parumcincta*), but suggested a closer relationship of *M. cartusiana* and *M. claustralis* to Italian *M. parumcincta* than to Corfu *M. parumcincta*.

**Figure 20.**
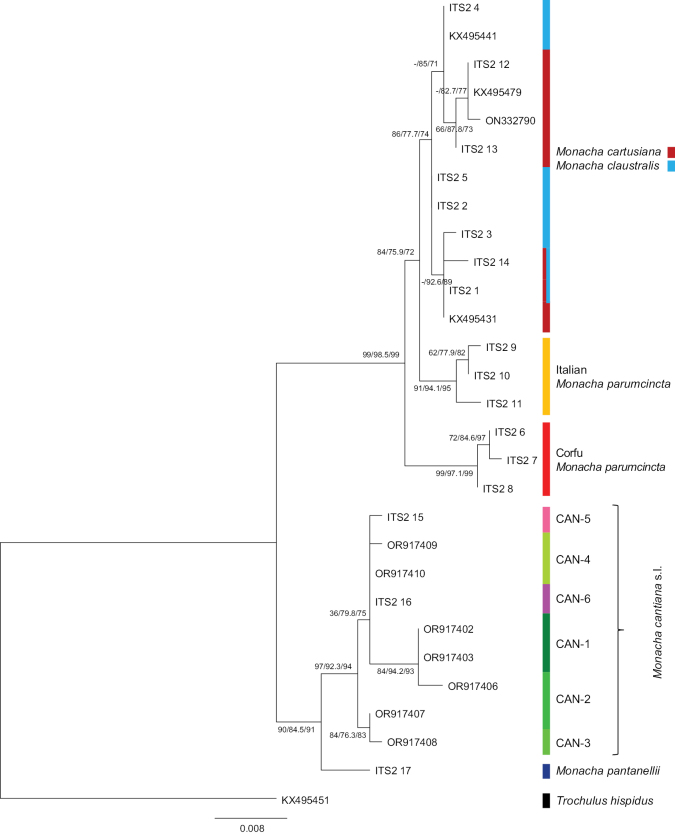
Maximum Likelihood (ML) tree of ITS2 (flanked with 5.8S and 28SrDNA) haplotypes of *Monacha claustralis* and *M. parumcincta* from Corfu (Kérkyra). ITS2 sequences of *M. claustralis* and *M. parumcincta* from Corfu (Table 1) were compared with new ITS2 sequences of *M. cartusiana*, Italian *M. parumcincta*, *M. pantanellii* and *M. cantiana* s.l., as well as with ITS2 sequences of *M. claustralis* and *M. cantiana* s.l. obtained from GenBank (Tables 1, 2). Numbers next to main branches indicate (left to right): bootstrap supports above 50% calculated by ML-MEGA7 (Kumar et al. 2016) on 1000 replicates (Felsenstein 1985), and SH-aLRT and ultrafast bootstrap in IQ-Tree (Nguyen et al. 2015). The tree was rooted with *Trochulus hispidus* sequences from GenBank (Table 2).

Analysis of three fragments of nuclear genes (5.8SrDNA, ITS2, 28SrDNA) confirmed the separateness of Italian *M. parumcincta* and Corfu *M. parumcincta* and between them and all the other species analysed (Fig. 20). However these gene fragments did not differentiate *M. claustralis* from *M. cartusiana*. The fragments of nuclear genes clustered together in one clade.

**Figure 21.**
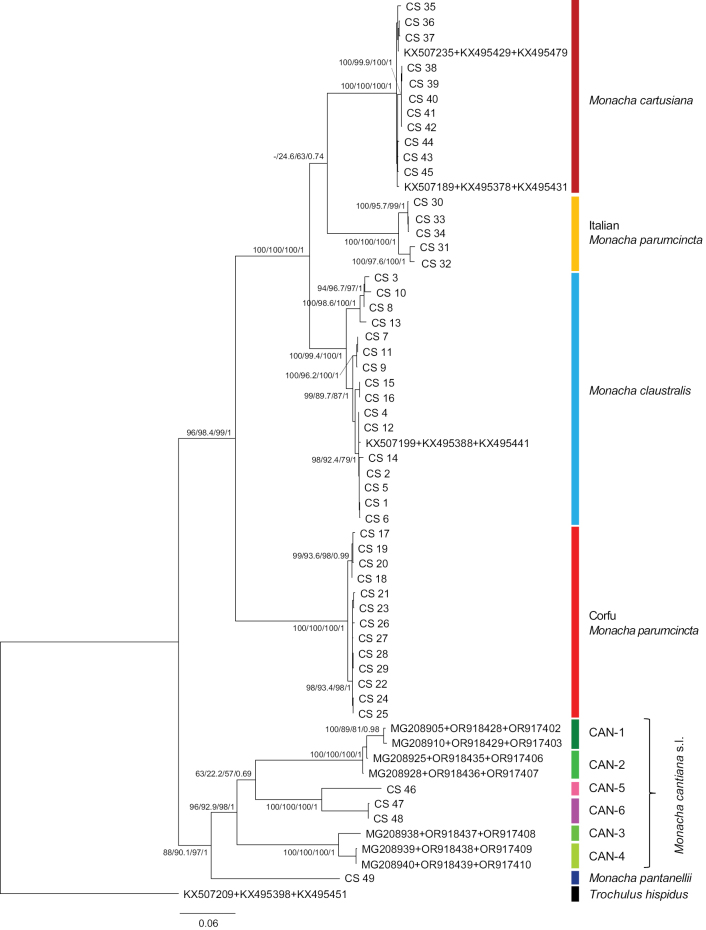
Maximum Likelihood (ML) tree of concatenated sequences of COI+16SrDNA+ITS2 (flanked with 5.8S and 28SrDNA) haplotypes of *Monacha claustralis* and *M. parumcincta* from Corfu (Kérkyra). Concatenated COI, 16SrDNA and ITS2 sequences of *M. claustralis* and *M. parumcincta* from Corfu (Table 1) were compared with new sequences of these gene fragments of *M. cartusiana*, Italian *M. parumcincta*, *M. pantanellii* and *M. cantiana* s.l. together with sequences obtained from GenBank (Tables 1, 2). Numbers next to main branches indicate (left to right): bootstrap supports above 50% calculated by ML-MEGA7 (Kumar et al. 2016) on 1000 replicates (Felsenstein 1985), SH-aLRT and ultrafast bootstrap in IQ-Tree (Nguyen et al. 2015), and posterior probabilities by BI (Ronquist et al. 2012). The tree was rooted with *Trochulus hispidus* concatenated sequences obtained from GenBank (Table 2).

The phylogenetic tree of concatenated sequences COI+16SrDNA+ITS2 (flanked with 5.8S and 28SrDNA) was similar in ML analysis with MEGA7 and IQ-Tree software and similar in Bayesian analysis. The phylogenetic tree of concatenated mitochondrial and nuclear gene sequences (Fig. 21, Table 2) showed separate clades for four species (*M. cartusiana*, Italian *M. parumcincta*, *M. claustralis*, and Corfu *M. parumcincta*), which were also separate from sequences of *M. pantanellii* and six lineages of *M. cantiana* sensu lato.

## ﻿Discussion

This research enabled us to characterise the topotypical populations of *Monacha claustralis* with respect to *Monacha cartusiana*, and those of *Monacha parumcincta* with respect to the Italian populations currently assigned to this species, on a morphological and molecular basis.

No distinction based on shell features was possible between *Monacha claustralis* and *Monacha cartusiana* either by qualitative examination or morphometric analysis. The absence of diagnostic shell characters is consistent with previous studies (Hausdorf 2000a, 2000b; Pieńkowska et al. 2015, 2018a; Williams et al. 2024). Conversely, distinction on the basis of genital characters was possible and the main qualitative differences between these species concerned the distal vagina, lateral vaginal sac, and vaginal appendix (for details see Results: Figs 8A, 9A, 10A for *M. claustralis* vs Fig. 11A, D for *M. cartusiana*; see also Hausdorf 2000a: fig. 19 for *M. claustralis* vs Hausdorf 2000b: fig. 13 for *M. cartusiana*; Pieńkowska et al. 2015: figs 13, 14 for *M. claustralis* vs figs 11, 12 for *M. cartusiana*; Pieńkowska et al. 2018a: figs 4, 5 for *M. claustralis* vs figs 6–8 for *M. cartusiana*). Quantitative differences were also revealed by morphometric analysis and LDA, which confirmed that ratios strongly linked to the female genitalia (V/VA and DBC/V) are the most effective parameters for taxonomic discrimination. Other supporting metrics, such as δ and standard distances, suggest that these differences are predominantly due to variations in shape rather than size (Fig. 12, Table 5). Interestingly, the discriminant ratios Ep/P and Ep/V_gm_, reported as effective for differentiating most individuals in reference populations of *M. claustralis* and *M. cartusiana* (Hausdorf 2000a, 2000b; Williams et al. 2024), did not prove significant in our study, at least in the dataset analysed. This discrepancy could reflect genuine differences between the populations examined in the different studies, or alternatively variations introduced by different sexual maturity or specimen fixation methods, or even different ways of measuring. For example, specimens studied by Williams et al. (2024) were fixed after being killed instantly in boiling water, those studied by Pieńkowska et al. (2015, 2018a) were fixed after being killed by drowning. Measurements were always made directly on isolated genitalia by Pieńkowska et al. (2015, 2018a), sometimes on the published figures of genitalia by Williams et al. (2024). Since the sections measured differed only minimally, different fixation protocols or measurement methods could introduce artificial deviations.

The distinctiveness of *M. claustralis* from other species of the genus *Monacha* is confirmed by our analysis of the nucleotide sequences of selected genes (Figs 18–21). It is particularly evident in the case of analysis of mitochondrial genes (COI and 16SrDNA: Figs 18, 19, respectively). It is noteworthy that sometimes analysis of morphological features and mitochondrial sequences was not consistent (Sauer and Hausdorf 2010, 2012). However, in our case, anatomical results and mitochondrial sequences support the distinctness of *M. claustralis* and *M. cartusiana*.

For nuclear genes (ITS2 with fragments of the flanking genes 5.8S and 28SrDNA: Fig. 20), the sequences obtained from Corfu *M. claustralis* specimens grouped in a separate clade from those of *M. parumcincta* from Corfu and Italy and from the sequences obtained for specimens representing the lineages of *M. cantiana* s.l. studied. However, the sequences of these nuclear genes did not distinguish *M. claustralis* from *M. cartusiana*. Moreover, one specimen (Ben1), found on Corfu, had an ITS2 sequence identical to *M. cartusiana* specimens from Italy (Quattrovie: Que1 – Que5). A similar situation with identical ITS2 sequence in specimens identified anatomically and by mitochondrial sequences as *M. claustralis* and *M. cartusiana* was previously encountered in Prague (Pieńkowska et al. 2015). Nuclear gene fragments also weakly supported the separateness of some *M. cantiana* s.l. lineages (Pieńkowska et al. 2018b, 2024). However, it is worth noting that sometimes ITS2 gene sequences are eliminated from phylogenetic analysis (Madeira et al. 2010).

Specimens of doubtful or uncertain attribution have been reported in non-native populations of *M. claustralis* and *M. cartusiana* from central and eastern Europe (Čejka et al. 2020; Gural-Sverlova and Gural 2022, 2023; Lesicki et al. 2024; Williams et al. 2024). Čejka et al. (2020) also described the vaginal sac in alleged specimens of *Monacha claustralis* from the Czech Republic, concluding that *Monacha cartusiana* and *Monacha claustralis* “in Central Europe possibly represent lineages of the same species derived from different parts of its native range”. Gural-Sverlova and Gural (2022, 2023) and Williams et al. (2024) described variations in the vaginal sac and/or overlapping genital ratios and suggested the possibility that the two species may hybridise. Lesicki et al. (2024) found specimens with divergent anatomical and molecular identification, i.e. some specimens had the reproductive structure of *M. cartusiana* and mtDNA of *M. claustralis*, others recognised anatomically as *M. claustralis* showed *M. cartusiana* haplotypes, while further specimens with *M. cartusiana* or *M. claustralis* haplotypes were characterised by an unusual female part of the reproductive system (moderately long vagina with slight diverticula in various places). These specimens are probably hybrids in which the mitochondrial genome, introduced by the egg cell, retains the features of one or the other species, while anatomical structure and nuclear genes have variably pronounced intermediate features (Lesicki et al. 2024). Current knowledge requires caution in using the results of our earlier papers (Pieńkowska et al. 2015, 2018a), which were written at a time when we were not aware of the possible hybridisation of *M. claustralis* and *M. cartusiana*. Moreover, these species are constantly expanding their ranges with larger overlaps, thus increasing the possibility of cross-breeding (Gural-Sverlova and Gural 2022, 2023; Lesicki et al. 2024; Williams et al. 2024).

Apart from minor differences in opacity and in the presence of whitish peripheral and subsutural bands, no distinction based on shell features was possible between the Corfu populations of *Monacha parumcincta* and the Italian ones currently assigned to this species. Indeed the morphometric analysis showed that neither the size nor the ratios clearly separated the two groups of populations assigned to *Monacha parumcincta*. This explains why, in the absence of anatomical and molecular evidence, they were previously considered conspecific (e.g. Forcart 1965; Manganelli et al. 1995; Pieńkowska et al. 2018b).

On the contrary, even in this case, distinction on the basis of genital characters, whether qualitative or morphometric, was clear. The most evident differences regarding the vagina, vaginal appendix, and transverse section of central duct of penial papilla (for details see Results: Figs 13A, 14A, 15A in Corfu populations vs Fig. 16A, D in Italian populations).

Morphometric analysis confirmed that the ratios identified by LDA are appropriate for distinguishing the groups. The primary discriminating ratio, E/VA, is driven primarily by shape differences rather than size, as indicated by its δ value close to zero. This suggests that separation is based on genuine morphological differences, with minimal influence from allometry. Such a conclusion is further supported by the high standard distance and the distinct, minimally overlapping ranges observed between the two taxa. A secondary ratio, F/V, while slightly less powerful, complemented E/VA by providing an additional dimension of discrimination.

The sequences of mitochondrial and nuclear gene fragments analysed created two separate groups for *M. parumcincta* from Corfu and *M. parumcincta* from Italy (Figs 18–21). They were also separate from sequences of all the other *Monacha* species analysed (i.e. *M. claustralis*, *M. cartusiana*, *M. cantiana* s.l., and *M. pantanellii*); this separateness was also supported by the analysis of nuclear genes. The separateness of Corfu and Italian *M. parumcincta* was further supported by K2P distances (Table 7), the mean value of which was 19.4%. However, in the case of gastropods, the genetic distances of COI sequences may not be decisive for species distinctiveness (see discussion on the necessary caution needed in drawing taxonomic conclusions based on COI sequences in our earlier papers: Pieńkowska et al. 2018b, 2019, 2020). Nevertheless we underline that all the data collected proves that the Italian populations assigned to *Monacha parumcincta* differ considerably, both morphologically and molecularly, from Corfu populations of *M. parumcincta*, and must be attributed to a different species.

## ﻿Additional information

### Use of AI

No use of AI was reported.

### Author ORCIDs

Giuseppe Manganelli https://orcid.org/0000-0002-8453-280X

Joanna R. Pieńkowska https://orcid.org/0000-0003-0372-121X

Debora Barbato https://orcid.org/0000-0003-1105-1711

Andrea Benocci https://orcid.org/0000-0003-3145-215X

Katarzyna Sosnowska https://orcid.org/0000-0002-7506-4231

Roy Anderson https://orcid.org/0009-0005-8420-3984

Folco Giusti https://orcid.org/0000-0001-8722-4653

Andrzej Lesicki https://orcid.org/0000-0002-1924-1934

### Data availability

All of the data that support the findings of this study are available in the main text.

## References

[B1] Bank RA Neubert E (2017) Checklist of the land and freshwater Gastropoda of Europe. MolluscaBase source details. https://www.molluscabase.org [Last update 16 July 2017]

[B2] Baur H Kranz-Baltensperger Y Cruaud A Rasplus J-Y Timokhov AV Gokhman VE (2014) Morphometric analysis and taxonomic revision of *Anisopteromalus* Ruschka (Hymenoptera: Chalcidoidea: Pteromalidae) – an integrative approach. Systematic Entomology 39(4): 691–709. 10.1111/syen.12081 26074661 PMC4459240

[B3] Baur H Leuenberger C (2011) Analysis of ratios in multivariate morphometry. Systematic Biology 60(6): 813–825. 10.1093/sysbio/syr061 21828084 PMC3193766

[B4] Baur H Leuenberger C (2020) Multivariate Ratio Analysis (MRA): R-scripts and tutorials for calculating Shape PCA, Ratio Spectra and LDA Ratio Extractor. Zenodo. https://zenodo.org/records/4250142 [accessed 17 May 2025]

[B5] BioEdit (2017) BioEdit 7.2. https://bioedit.software.informer.com/7.2 [accessed 15 April 2024]

[B6] Caro A Neiber MT Gomez-Moliner BJ Madeira MJ (2019) Molecular phylogeny and biogeography of the land snail subfamily Leptaxinae (Gastropoda: Hygromiidae). Molecular Phylogenetics and Evolution 139: 106570. 10.1016/j.ympev.2019.106570 31349101

[B7] Čejka T Beran L Korábek O Hlaváč JČ Horáčková L Coufal R Drvotová M Maňas M Horsáková V Horsák M (2020) Malacological news from the Czech and Slovak Republics in 2015–2019. Malacologica Bohemoslovaca 19: 71–106. 10.5817/MaB2020-19-71

[B8] Chernomor O von Haeseler A Minh BQ (2016) Terrace aware data structure for phylogenomic inference from supermatrices. Systematic Biology 65(6): 997–1008. 10.1093/sysbio/syw037 27121966 PMC5066062

[B9] Chiba S (1999) Accelerated evolution of land snails *Mandarina* in the oceanic Bonin Islands: Evidence from mitochondrial DNA sequences. Evolution 53(2): 460–471. 10.2307/2640782 28565404

[B10] Dabert M Witalinski W Kazmierski A Olszanowski Z Dabert J (2010) Molecular phylogeny of acariform mites (Acari, Arachnida): Strong conflict between phylogenetic signal and long-branch attraction artifacts. Molecular Phylogenetics and Evolution 56(1): 222–241. 10.1016/j.ympev.2009.12.020 20060051

[B11] De Stefani C (1879) Nuove specie di molluschi viventi nell’Italia centrale. Bullettino della Società Malacologica Italiana 5(1/3): 38–48. https://www.biodiversitylibrary.org/item/120073#page/7/mode/1up

[B12] Eddy S (1998) Profile hidden Markov models. Bioinformatics 14(9): 755–763. 10.1093/bioinformatics/14.9.755 9918945

[B13] Felsenstein J (1985) Confidence limits on phylogenies: An approach using the bootstrap. Evolution 39(4): 783–791. 10.2307/2408678 28561359

[B14] Fitzinger LJ (1833) Systematisches Verzeichniß der im Erzherzogthume Oesterreich vorkommenden Weichthiere, als Prodrom einer Fauna derselben. Beiträge zur Landeskunde Oesterreichs’s unter der Enns 3: 88–122. [Wien] https://biodiversitylibrary.org/page/10601570

[B15] Folmer O Black M Hoeh W Lutz RA Vrijenhoek RC (1994) DNA primers for amplification of mitochondrial cytochrome c oxidase subunit I from diverse metazoan invertebrates. Molecular Marine Biology and Biotechnology 3: 294–299. 7881515

[B16] Forcart L (1965) Rezente Land- und Süsswassermollusken der süditalienischen Landschaften Apulien, Basilicata und Calabrien. Verhandlungen der naturforschenden Gesellschaft in Basel 76(1): 59–184.

[B17] Glez-Peña D Gómez-Blanco D Reboiro-Jato M Fdez-Riverola F Posada D (2010) ALTER: Program-oriented conversion of DNA and protein alignments. Nucleic Acids Research 38(suppl. 2): W14–W18. 10.1093/nar/gkq321 PMC2896128 20439312

[B18] Guindon S Dufayard J-F Lefort V Anisimova M Hordijk W Gascuel O (2010) New algorithms and methods to estimate maximum-likelihood phylogenies: Assessing the performance of PhyML 3.0. Systematic Biology 59(3): 307–321. 10.1093/sysbio/syq010 20525638

[B19] Gural-Sverlova N Gural R (2022) *Monacha claustralis* i *M. cartusiana* (Gastropoda, Hygromiidae) – dva kripticheskih vida antropokhornych nazemnych mollyuskov na zapade Ukrainy. Ruthenica 32: 69–80. 10.35885/ruthenica.2022.32(2).3 [*Monacha claustralis* and *M. cartusiana* (Gastropoda, Hygromiidae), two cryptic species of anthropochorous land molluscs in Western Ukraine]

[B20] Gural-Sverlova N Gural R (2023) Three introduced *Monacha* species (Gastropoda: Hygromiidae) in and near Lviv with remarks on *M. cartusiana* spreading in Ukraine and its Western part. Folia Malacologica 31(2): 69–82. 10.12657/folmal.031.012

[B21] Hall TA (1999) BioEdit: A user friendly biological sequence alignment editor and analysis program for Windows 95/98/NT. Nucleic Acids Symposium Series 41: 95–98.

[B22] Hasegawa M Kishino H Yano T (1985) Dating the human-ape split by a molecular clock of mitochondrial DNA. Journal of Molecular Evolution 22(2): 160–174. 10.1007/BF02101694 3934395

[B23] Hausdorf B (2000a) The genus *Monacha* in Turkey (Gastropoda: Pulmonata: Hygromiidae). Archiv fur Molluskenkunde International Journal of Malacology 128(1–2): 61–151. 10.1127/arch.moll/128/2000/61

[B24] Hausdorf B (2000b) The genus *Monacha* in the Western Caucasus (Gastropoda: Hygromiidae). Journal of Natural History 34(8): 1575–1594. 10.1080/00222930050117495

[B25] Hesse P (1914) *Helix frequens* Mousson (Helicidae, Mollusca). Mitteilungen des Kaukasischen Museums 6: 253–270 [1 pl].

[B26] Hoang DT Chernomor O von Haeseler A Minh BQ Vinh LS (2018) UFBoot2: Improving the ultrafast bootstrap approximation. Molecular Biology and Evolution 35(2): 518–522. 10.1093/molbev/msx281 29077904 PMC5850222

[B27] Hutchinson JMC Schlitt B Reise H (2019) *Monacha claustralis* (Rossmässler, 1834), a hygromiid snail new to Germany. Mitteilungen der Deutschen Malakozoologische Gesellschaft 100: 17–22.

[B28] Jukes TH Cantor CR (1969) Evolution of protein molecules. In: Munro HN (Ed.) Mammalian Protein Metabolism.Academic Press, New York, 21–132. 10.1016/B978-1-4832-3211-9.50009-7

[B29] Kalyaanamoorthy S Minh BQ Wong TKF von Haeseler A Jermiin LS (2017) ModelFinder: Fast model selection for accurate phylogenetic estimates. Nature Methods 14(6): 587–589. 10.1038/nmeth.4285 28481363 PMC5453245

[B30] Kimura M (1980) A simple method for estimating evolutionary rate of base substitutions through comparative studies of nucleotide sequences. Journal of Molecular Evolution 16(2): 111–120. 10.1007/BF01731581 7463489

[B31] Koetschan C Förster F Keller A Schleicher T Ruderisch B Schwarz R Müller T Wolf M Schultz J (2010) The ITS2 Database III - sequences and structures for phylogeny. Nucleic Acids Research 38(Suppl. 1): D275–D279. 10.1093/nar/gkp966 PMC2808966 19920122

[B32] Kumar S Stecher G Tamura K (2016) MEGA7: Molecular Evolutionary Genetics Analysis version 7.0 for bigger datasets. Molecular Biology and Evolution 33(7): 1870–1874. 10.1093/molbev/msw054 27004904 PMC8210823

[B33] Lesicki A Manganelli G Barbato D Pieńkowska JR Żurańska H Sosnowska K Proćków M Kuźnik-Kowalska E Giusti F (2024) Possible hybridisation of *Monacha cartusiana* (Müller, 1774) and *M. claustralis* (Rossmässler, 1834) in populations from Moldova, Romania, the Czech Republic and Poland. In: Marzec M (Еd.) The 38^th^ Polish Malacological Seminar, Seminar Report. Folia Malacologica 32(3): 209. 10.12657/folmal.032.019

[B34] Linnaeus C (1758) Systema naturæ per regna tria naturæ, secundum classes, ordines, genera, species, cum characteribus, differentiis, synonymis, locis. Editio decima, reformata. Tomus I. Salvius, Holmiæ, [4 +] 824 pp. 10.5962/bhl.title.559

[B35] Madeira MJ Elejalde MA Chueca LJ Gómez-Moliner BJ (2010) Phylogenetic position of the genus *Cryptazeca* and the family Azecidae within the system of the Stylommatophora. Malacologia 52(1): 163–168. 10.4002/040.052.0110

[B36] Manganelli G Bodon M Favilli L Giusti F (1995) GastropodaPulmonata. In: Minelli A, Ruffo S, La Posta S (Eds) Checklist delle specie della fauna d’Italia, Calderini, Bologna 16: 1–60.

[B37] Minh BQ Nguyen MAT von Haeseler A (2013) Ultrafast approximation for phylogenetic bootstrap. Molecular Biology and Evolution 30(5): 1188–1195. 10.1093/molbev/mst024 23418397 PMC3670741

[B38] MolluscaBase (2024) *Monacha* Fitzinger, 1833. MolluscaBase. https://www.molluscabase.org/aphia.php?p=taxdetails&id=426406 [accessed 21 Dec 2024]

[B39] Montagu G (1803) Testacea Britannica, or, Natural history of British shells, marine, land, and fresh-water, including the most minute: systematically arranged and embellished with figures. 2 volumes, Romsey, London, [xxxvii +] 606 pp. 10.5962/bhl.title.33927

[B40] Müller OF (1774) Vermium terrestrium et fluviatilium, seu animalium infusiorium, helminthicorum, et testaceorum, non marinorum, succinct historia. Vol. II. Heineck & Faber, Havniae et Lipsiae, [xxxvi + 214 +] 10 pp. 10.5962/bhl.title.46299

[B41] Nei M Kumar S (2000) Molecular evolution and phylogenetics. Oxford University Press, Oxford New York, 352 pp. 10.1093/oso/9780195135848.001.0001

[B42] Neiber MT Hausdorf B (2015) Phylogeography of the land snail genus *Circassina* (Gastropoda: Hygromiidae) implies multiple Pleistocene refugia in the western Caucasus region. Molecular Phylogenetics and Evolution 93: 129–142. 10.1016/j.ympev.2015.07.012 26220841

[B43] Neiber MT Hausdorf B (2017) Molecular phylogeny and biogeography of the land snail genus *Monacha* (Gastropoda, Hygromiidae). Zoologica Scripta 46(3): 308–321. 10.1111/zsc.12218

[B44] Neiber MT Razkin O Hausdorf B (2017) Molecular phylogeny and biogeography of the land snail family Hygromiidae (Gastropoda: Helicoidea). Molecular Phylogenetics and Evolution 111: 169–184. 10.1016/j.ympev.2017.04.002 28390908

[B45] Nguyen L-T Schmidt HA von Haeseler A Minh BQ (2015) IQ-TREE: A fast and effective stochastic algorithm for estimating maximum likelihood phylogenies. Molecular Biology and Evolution 32(1): 268–274. 10.1093/molbev/msu300 25371430 PMC4271533

[B46] Pieńkowska JR Giusti F Manganelli G Lesicki A (2015) *Monacha claustralis* (Rossmässler 1834) new to Polish and Czech malacofauna (Gastropoda: Pulmonata: Hygromiidae). Journal of Conchology 42(1): 79–93.

[B47] Pieńkowska JR Proćków M Górka M Lesicki A (2018a) Distribution of *Monacha claustralis* (Rossmässler, 1834) and *M. cartusiana* (O. F. Müller, 1774) (Eupulmonata: Hygromiidae) in central European and Balkan countries: new data. Folia Malacologica 26(2): 103–120. 10.12657/folmal.026.009

[B48] Pieńkowska JR Manganelli G Giusti F Hallgass A Lesicki A (2018b) Exploring *Monacha cantiana* (Montagu, 1803) phylogeography: Cryptic lineages and new insights into the origin of the English populations (Eupulmonata, Stylommatophora, Hygromiidae). ZooKeys 765: 1–41. 10.3897/zookeys.765.24386 PMC5999686 29904267

[B49] Pieńkowska JR Manganelli G Giusti F Barbato D Hallgass A Lesicki A (2019) Exploration of phylogeography of *Monacha cantiana* s.l. continues: The populations of the Apuan Alps (NW Tuscany, Italy) (Eupulmonata, Stylommatophora, Hygromiidae). ZooKeys 814: 115–149. 10.3897/zookeys.814.31583 PMC6335383 30655712

[B50] Pieńkowska JR Manganelli G Giusti F Barbato D Kosicka E Hallgass A Lesicki A (2020) Redescription of *Monacha pantanellii* (De Stefani, 1879), a species endemic to the central Apennines, Italy (Gastropoda, Eupulmonata, Hygromiidae) by an integrative molecular and morphological approach. ZooKeys 988: 17–61. 10.3897/zookeys.988.56397 33223890 PMC7666099

[B51] Pieńkowska JR Manganelli G Proćków M Gürelli G Kosicka E Giusti F Lesicki A (2022) *Monacha samsunensis* (Pfeiffer, 1868): another Anatolian species introduced to Western Europe, where it is known as *Monacha atacis* Gittenberger & de Winter, 1985 (Gastropoda: Eupulmonata: Hygromiidae). The European Zoological Journal 89(1): 966–990. 10.1080/24750263.2022.2100932

[B52] Pieńkowska JR Manganelli G Proćków M Barbato D Sosnowska K Giusti F Lesicki A (2024) Next step in *Monacha cantiana* (Montagu, 1803) phylogeography: Northern French and Dutch populations (Eupulmonata, Stylommatophora, Hygromiidae). ZooKeys 1198: 55–86. 10.3897/zookeys.1198.119738 38693970 PMC11061557

[B53] Pilsbry HA (1894) Manual of Conchology; structural and systematic. With illustrations of the species. Second Series: Pulmonata. Vol. 9 (Helicidæ, Vol. 7). Guide to the study of helices. Issue 36, pp. 161–366, pls 41–71, frontispiece. Published by Conchological Section, Academy of Natural Sciences, Philadelphia.

[B54] R Core Team (2021) R: A language and environment for statistical computing. R Foundation for Statistical Computing, Vienna, Austria. https://www.R-project.org/ [accessed 15 September 2024]

[B55] Ronquist F Teslenko M van der Mark P Ayres DL Darling A Höhna S Larget B Liu L Suchard MA Huelsenbeck JP (2012) MrBayes 3.2: Efficient Bayesian phylogenetic inference and model selection across a large model space. Systematic Biology 61(3): 539–542. 10.1093/sysbio/sys029 22357727 PMC3329765

[B56] Rossmässler EA (1834) Diagnoses conchyliorum terrestrium et fluviatilium. Zugleich zu Fascikeln natürlicher Exemplare. II Heft. No. 21–40. Arnold, Dresden & Leipzig, 8 pp. 10.5962/bhl.title.10380

[B57] Sauer J Hausdorf B (2010) Reconstructing the evolutionary history of the radiation of the land snail genus *Xerocrassa* on Crete based on mitochondrial sequences and AFLP markers. BMC Evolutionary Biology 10(1): 299. 10.1186/1471-2148-10-299 20920353 PMC2958919

[B58] Sauer J Hausdorf B (2012) A comparison of DNA-based methods for delimiting species in a Cretan land snail radiation reveals shortcomings of exclusively molecular taxonomy. Cladistics 28(3): 300–316. 10.1111/j.1096-0031.2011.00382.x 34872193

[B59] Tamura K (1992) Estimation of the number of nucleotide substitutions when there are strong transition-transversion and G + C-content biases. Molecular Biology and Evolution 9(4): 678–687. 10.1093/oxfordjournals.molbev.a040752 1630306

[B60] Tavaré S (1986) Some probabilistic and statistical problems in the analysis of DNA sequences. In: Miura RM (Еd.). Some mathematical questions in biology - DNA sequence analysis. Lectures on Mathematics in the Life Sciences vol. 17. The American Mathematical Society, Providence, RI, 57–86.

[B61] Thompson JD Higgins DG Gibson TJ (1994) CLUSTAL W: Improving the sensitivity of progressive multiple sequence alignment through sequence weighting, position specific gap penalties and weight matrix choice. Nucleic Acids Research 22(22): 4673–4680. 10.1093/nar/22.22.4673 7984417 PMC308517

[B62] Wade CM Mordan PB (2000) Evolution within the gastropod molluscs; using the ribosomal RNA gene-cluster as an indicator of phylogenetic relationships. The Journal of Molluscan Studies 66(4): 565–570. 10.1093/mollus/66.4.565

[B63] Welter-Schultes FW (2012) European non-marine molluscs, a guide for species identification. Planet Poster Editions, Göttingen, 679 pp.

[B64] Williams BMJ Hutchinson JMC Reise H Zauder O Schlitt B (2024) Difficulties in distinguishing *Monacha claustralis* from *M. cartusiana* in Germany and Poland. The Journal of Molluscan Studies 90(3): eyae030. 10.1093/mollus/eyae030

